# Enabling Long Cycle Life and High Rate Iron Difluoride Based Lithium Batteries by In Situ Cathode Surface Modification

**DOI:** 10.1002/advs.202201419

**Published:** 2022-05-14

**Authors:** Yong Su, Jingzhao Chen, Hui Li, Haiming Sun, Tingting Yang, Qiunan Liu, Satoshi Ichikawa, Xuedong Zhang, Dingding Zhu, Jun Zhao, Lin Geng, Baiyu Guo, Congcong Du, Qiushi Dai, Zaifa Wang, Xiaomei Li, Hongjun Ye, Yunna Guo, Yanshuai Li, Jingming Yao, Jitong Yan, Yang Luo, Hailong Qiu, Yongfu Tang, Liqiang Zhang, Qiao Huang, Jianyu Huang

**Affiliations:** ^1^ School of Materials Science and Engineering Xiangtan University Xiangtan Hunan 411105 P. R. China; ^2^ Clean Nano Energy Center State Key Laboratory of Metastable Materials Science and Technology Yanshan University Qinhuangdao 066004 P. R. China; ^3^ Research Center for Ultra‐High Voltage Electron Microscopy Osaka University Ibaraki Osaka 567‐0047 Japan

**Keywords:** conversion cathodes, electrolyte decomposition, lithium‐ion batteries, long cycle lifetime, stable Fe_3_O_4_ layers

## Abstract

Metals fluorides (MFs) are potential conversion cathodes to replace commercial intercalation cathodes. However, the application of MFs is impeded by their poor electronic/ionic conductivity and severe decomposition of electrolyte. Here, a composite cathode of FeF_2_ and polymer‐derived carbon (FeF_2_@PDC) with excellent cycling performance is reported. The composite cathode is composed of nanorod‐shaped FeF_2_ embedded in PDC matrix with excellent mechanical strength and electronic/ionic conductivity. The FeF_2_@PDC enables a reversible capacity of 500 mAh g^–1^ with a record long cycle lifetime of 1900 cycles. Remarkably, the FeF_2_@PDC can be cycled at a record rate of 60 C with a reversible capacity of 107 mAh g^–1^ after 500 cycles. Advanced electron microscopy reveals that the in situ formation of stable Fe_3_O_4_ layers on the surface of FeF_2_ prevents the electrolyte decomposition and leaching of iron (Fe), thus enhancing the cyclability. The results provide a new understanding to FeF_2_ electrochemistry, and a strategy to radically improve the electrochemical performance of FeF_2_ cathode for lithium‐ion battery applications.

## Graphical abstract

Embedding nano‐FeF_2_ into polymer derived carbon (FeF_2_@PDC) matrix with excellent mechanical strength and electron/ion conductivity enables in situ formation of a Fe_3_O_4_ layer that suppresses the decomposition of the electrolyte and inhibits the formation of a detrimental cathode solid electrolyte interface. The Li‐ FeF_2_@PDC cell exhibits a remarkable long cycle life of 1900 cycles with a reversible capacity over 500 mAh g^‐1^ at 0.5 C.

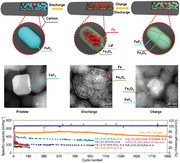

## 1 Introduction

Lithium‐ion batteries (LIBs) have become a major electrochemical energy storage technology for applications in transportations such as electric vehicles and grid energy storage,^[^ ^1^ ^]^ which demand LIBs with higher energy density with guaranteed safety.^[^ ^2^ ^]^ State‐of‐the‐art intercalation‐type cathodes have reached their theoretical capacity limit. Conversion‐type cathodes such as metal fluorides (MFs) have attracted significant interests in the battery community due to their outstanding gravimetric and volumetric capacities compared to the intercalation cathodes.^[^ ^3^ ^]^ The iron (Fe)‐based fluorides were of particular interests due to their high energy density, the abundance of Fe in the earth's crust, and possible lower cost.^[^ ^4^ ^]^

Despite multiple advantages, MFs suffer from capacity decay, low‐rate capability, and short cycle lifetime originated from undesirable electrolyte decomposition, poor electronic and ionic conductivity.^[^ ^5^ ^]^ The uncontrollable electrolyte decomposition leads to the detrimental cathode solid electrolyte interface (CEI) formation. The mechanical strength,^[^ ^6^ ^]^thickness and uniformity,^[^ ^6a^ ^]^ composition and stability^[^ ^6a^ ^]^ and the capability of fast Li^+^ transport^[^ ^7^ ^]^ of CEI impact significantly the stability of MFs cathode. A favorable CEI is able to hinder dissolution of active species,^[^ ^6^, ^8^ ^]^ while unstable CEI formation consumes electrolyte continuously, which shortens the battery's life.^[^ ^9^ ^]^ The CEI comprised mainly of organic/inorganic compounds decomposes upon charging.^[^ ^5^, ^8^ ^]^Although optimization of electrolyte^[^ ^6^, ^8^, ^10^ ^]^ and oxides deposition^[^ ^11^ ^]^ have been proved to be beneficial to the construction of favorable CEIs, the achieved cycle life of MFs was limited. Electrolyte decomposition and related detrimental CEI formation are bottlenecks to the electrochemical performance of MFs cathodes.

**Figure 1:**
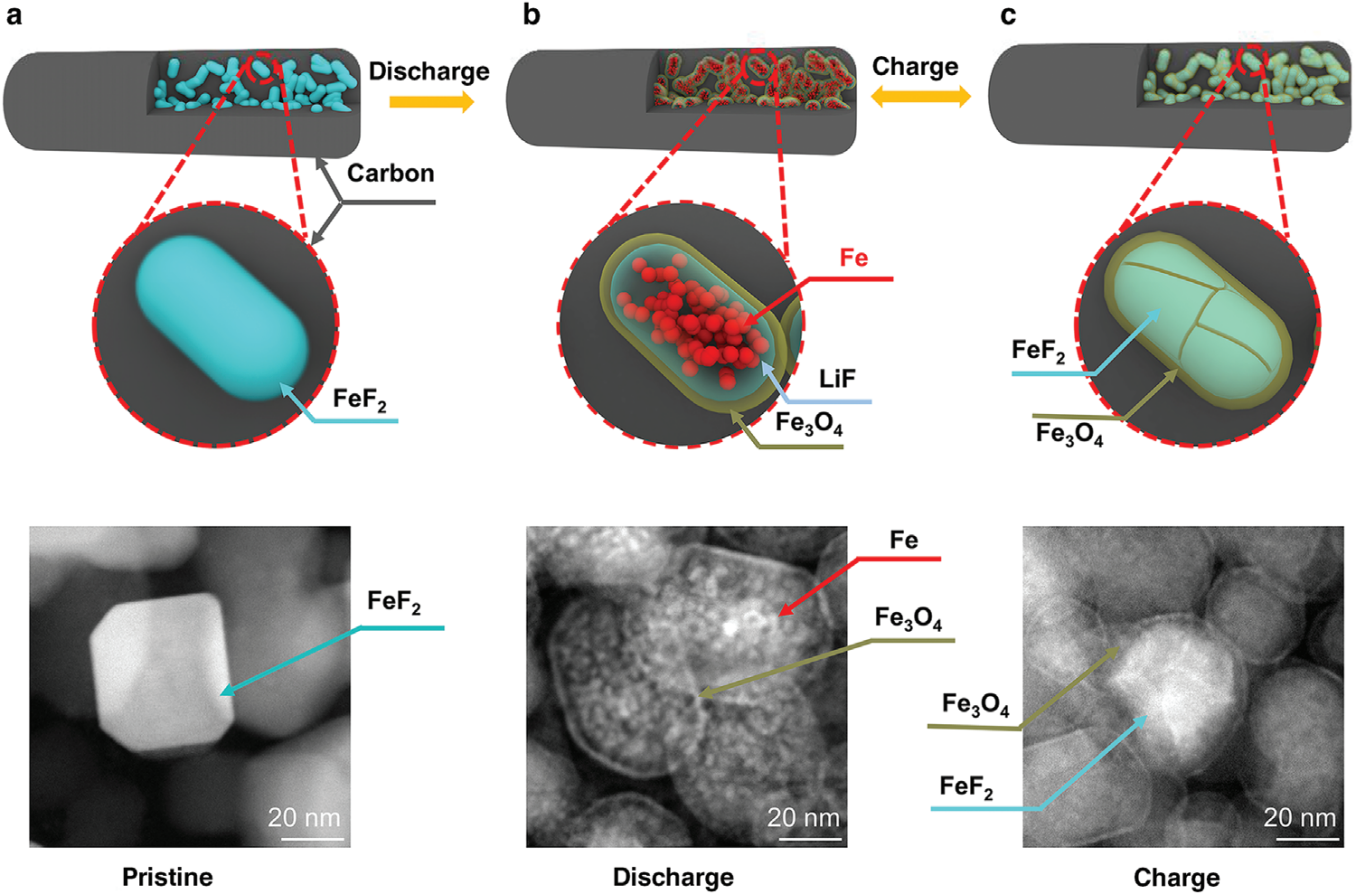
Schematic and the corresponding High‐angle annular dark‐field (HAADF) images of the FeF_2_@PDC composite cathode. a), FeF_2_ nanoparticles were embedded into a PDC matrix with high mechanical strength, mixed electronic and ionic conductivity. b), During discharge, FeF_2_ converted to interconnected speckle‐like Fe nanoparticles (bright contrast) with LiF (dark contrast) interlaced in between. A Fe_3_O_4_ layer with a thickness of 2 nm was formed on the outer surface of the active materials. c), Fe and LiF reconverted to FeF_2_ with the remaining of Fe_3_O_4_ layer during charge. The formation of a uniform Fe_3_O_4_ layer on the surface of the active materials effectively prevented the deleterious cathode/electrolyte interfacial reaction, the electrolyte decompositionand the dissolution of elemental Fe into the electrolyte.

Herein, we demonstrate that the roadblocks in FeF_2_ cathode can be removed by a novel composite structure design. By embedding nano‐FeF_2_ into polymer derived carbon (FeF_2_@PDC) matrix (**Figure** **1**a), the electrolyte decomposition and related undesirable CEI formation was suppressed. Advanced electron microscopy revealed the in situ formation of a Fe_3_O_4_ shell on the surface of the cathode active material (CAM) in the first discharge (Figure 1b) with excellent mechanical strength and passivation to liquid electrolyte after long cycles (Figure 1c). A remarkable long cycle life of 1900 cycles with a reversible capacity over 500 mAh g^–1^ at 0.5 C was achieved. Long cycle stability at ultrahigh rates of 30 C and 60 C with discharge capacities of 150 and 107 mAh g^–1^ after 500 cycles, respectively, was attained. Our results represent a new milestone in the FeF_2_ research and bring us one step closer toward the application of FeF_2_ cathode to enable high energy density LIBs.

## 2 Results and Discussion

### 2.1 Synthesis of FeF_2_@PDC Composite Cathode

**Figure 2:**
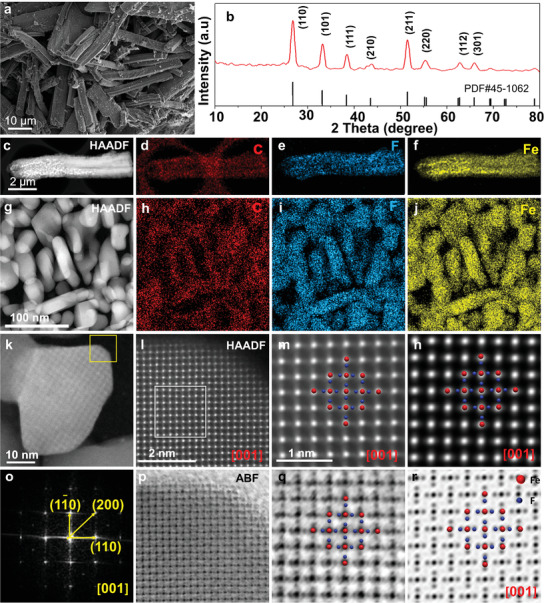
Morphological and structural analysis of the FeF_2_@PDC composite cathode. a), SEM image showing a micro‐rod like morphology with a diameter of 2 µm and a length of about 10 µm. b), XRD of the FeF_2_@PDC composites. c), HAADF image of a single FeF_2_@PDC microrod, and the corresponding (Energy dispersive X‐ray spectroscopy) EDX elemental mapping of d) C, e) F, and f) Fe. g) HAADF image of FeF_2_ particles (bright contrast) inside PDC (dark contrast), and the corresponding EDX elemental mapping of h) C, i) F, and j) Fe. k) HAADF image of a single FeF_2_ particle orientated at [001] zone axis. l), HAADF‐STEM image of an FeF_2_ particle (corresponding to the region enclosed by the yellow square in (k)). m), Higher‐magnification view corresponding to the region enclosed by a white square in (l) with the structural model overlaid in the image. n), Simulated HAADF‐STEM image of FeF_2_, which fits well with the experimental image in (m). o), FFT corresponding to (k) showing the FeF_2_ [001] zone axis. p), ABF‐STEM image corresponding to (l). q), Higher‐magnification ABF image corresponding to (m), showing each Fe atom is surrounded by four F atoms with two longer and two shorter bonds. r), Simulated ABF‐STEM image of FeF_2_, which fits well with the experiment image in (q).

A novel strategy was adopted (more details in the experimental section) to fabricate the FeF_2_@PDC composite cathode to ensure abundant electron/ion pathways.^[^ ^12^ ^]^ Note that among the various polymers (such as polyvinylidene difluoride (PVDF) or Polytetrafluoroethylene (PTFE) or polyvinyl alcohol (PVA)), only polyvinyl pyrrolidone (PVP) can dissolve well in ethanol, resulting in a transparent solution. Therefore, PVP was selected as the precursor to synthesize FeF_2_@PDC (Figure S1, Supporting Information). The FeF_2_@PDC composite consists of porous short rods with flat cross‐sections and length of 10–30 µm (**Figure** **2**a, c–f). The main pore size was about 4 nm (Figure S2, Supporting Information), and the porous structure permits the permeation of liquid electrolyte into the PDC bulk. Thermogravimetric analysis (TGA) shows that the weight loss of the precursor mixture was completed after 470°C (Figure S3, Supporting Information), suggesting that the formation of carbon with a low crystallinity at 550°C.^[^ ^13^ ^]^ Higher carbonization temperature resulted in undesirable side products (Figure S4, Supporting Information). The mass loading of FeF_2_ in the composite was evaluated to be about 74% (Figure S5, Supporting Information).

X‐ray diffraction (XRD) from FeF_2_@PDC shows a tetragonal FeF_2_ phase (JCPDS card no. 45–1062, Figure 2b) with absence of the PDC peak. The absence of PDC signal peaks in the FeF_2_@PDC sample is attributed to the low proportion and poor crystallinity of PDC. To further prove the existence of PDC material, X‐ray photoelectron spectroscopy (XPS) analysis of the pristine PVP and FeF_2_@PDC was conducted. According to the C 1s spectra from the pristine PVP (Figure S6a, Supporting Information), the peaks located at ≈285, 285.5, 286.5, and 288.3 eV correspond well to C—C, N—C═O, C—N, and C—C═O binding energies.^[^ ^14^ ^]^ After annealing, the C 1s spectra from the pristine FeF_2_@PDC show that the peaks located at ≈284.8, 286, and 288.8 eV correspond well to C—C, C—O, and C═O binding energies (Figure S6b, Supporting Information).^[^ ^15^ ^]^ The presence of oxygen (O) signals further indicates that the polymer is not highly carbonized. The rigidity of residual PDC with low crystallinity obtained after acid treatment (Figure S7, Supporting Information) was excellent, which may accommodate volume changes of FeF_2_ during cycling. High‐angle annular dark‐field‐scanning transmission electron microscopy (HAADF‐STEM) image with corresponding elemental mapping obtained from a thin foil/rod FeF_2_@PDC sample fabricated by focused ion beam (FIB) (Figure S8, Supporting Information) reveal that nanorod‐shaped FeF_2_ with an average diameter of 20 nm and length of 50 nm are evenly distributed in the PDC (Figure 2g–j). High resolution transmission electron microscopy (HRTEM) images show intimate contact between the FeF_2_ particle and the PDC (Figure S9, Supporting Information). The atomic structure of FeF_2_ was revealed by HAADF images (Figure 2k–m). The [001] FeF_2_ atomic image exhibits perfect crystallinity with rounded corner (Figure 2l). The simulated HAADF image (Figure 2n) matches excellently with the HAADF image (Figure 2l,m). The corresponding Fast Fourier Transform (FFT) confirms the FeF_2_ [001] zone axis (Figure 2o). Surface structure plays a vital role in CEI formation, and HAADF image shows that the FeF_2_ exhibits perfect surface structure without impurity. We successfully imaged FeF_2_ under annular bright‐field (ABF)‐STEM (Figure 2p,q). The positions of Fe and F atoms were precisely imaged with atomic resolution. The simulated ABF image (Figure 2r) matches excellently with the experimental one (Figure 2q). Imaging F atoms by ABF is significant^[^ ^16^ ^]^ in the context that it may help to clarify the formation of intermediates during the FeF_2_ conversion reaction, which is still under debate.^[^ ^8^, ^17^ ^]^ The above results demonstrate that a composite cathode comprising highly chemical purity FeF_2_ particles with uniform size and distribution in PDC (FeF_2_@PDC) was obtained.

### 2.2 Electrochemical Performances of the FeF_2_@PDC Composites Cathode

**Figure 3:**
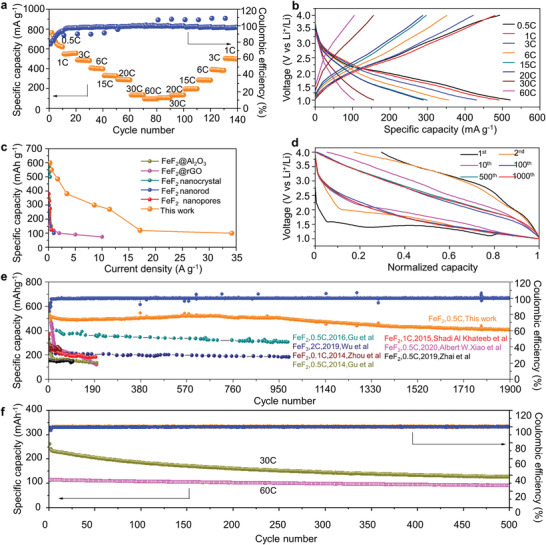
Electrochemical performance of FeF_2_@PDC/Li batteries cycled in 2 M LiFSI in DME. a), Rate performance of the FeF_2_@PDC cathode from 0.5 C to 60 C (1 C = 571 mAh g^–1^). b), Comparison of the rate performance for FeF_2‐_based electrodes. c), Charge and discharge profiles at different C‐rates. d), Charge and discharge profiles at 0.5 C. e), Long‐term cycling performance of the FeF_2_@PDC cathode at 0.5 C. f), Long cycle performance at ultrahigh rate of 30 C and 60 C. The voltage range was limited from 1 to 4 V (vs Li^+^/Li)

The electrochemical performances of the FeF_2_@PDC composites cathode were evaluated in 2 M lithium bis (fluorosulfonylimide) (LiFSI) in dimethoxyethane (DME) electrolyte over a voltage window of 1–4 V (vs Li^+^/Li), which is far lower than that in other studies.^[^ ^7^, ^18^ ^]^We intentionally lowered the salt concentration to test our hypotheses that the composite cathode may suppress the electrolyte decomposition. We indeed achieved outstanding electrochemical performance for the FeF_2_@PDC composite cathode even in significantly lower electrolyte concentration. Note that the capacity contribution from PDC was only 40 mAh g^–1^ (Figure S10, Supporting Information). Rate tests show that the composite cathode discharges from 0.5 C (1 C = 571 mAh g–1) with a capacity over 600 mAh g^–1^ to 60 C with a remarkable capacity of 107 mAh g^–1^. The cell discharges back to 1 C with a capacity of 525 mAh g^–1^, demonstrating the excellent rate capability and reversibility of the composite cathode (**Figure** **3**a). The charge/discharge curves at different rate also correlated with the discharge capacity in the rate tests (Figure 3b). The slightly higher than theoretical capacity observed at 0.5 C at the initial cycles may be attributed to the CEI formation on PDC or FeF_2_.^[^ ^6^ ^]^ The charge/discharge curves show that the voltage increases or decreases nearly monotonically with the capacity without apparent voltage plateaus, which is typical for FeF_2_ cathode in ether‐based electrolyte.^[^ ^6^, ^7^ ^]^ We further conducted cyclic voltammetry (CV) studies over the voltage window of 1–4V (vs Li^+^/Li) to elucidate the electrochemical conversion reaction mechanism (Figure S11, Supporting Information). The oxidation/reduction pair at 3.1V (OX1) and 1.75V (RE1) typically corresponds to the conversion reaction of FeF_2_, confirming the reversible electrochemical reactions.^[^ ^6^ ^]^ The oxidation/reduction pair peaks at 3.5V (OX2) and 3.0V (RE2) are likely related to the formation of Fe^3+^ species at the cathode surface.^[^ ^6^, ^9^, ^19^ ^]^

Note that MFs have poor Li^+^ transfer kinetics during cycling.^[^ ^20^ ^]^ The excellent rate capability is attributed to: 1) the nanosized FeF_2_ which shortens the electron and Li^+^ transfer distance and 2) the PDC wrapped around the FeF_2_ constitutes the electronic/ionic transport expressways for the nanosized FeF_2_, as carbon is an excellent mixed ionic and electronic conductor.^[^ ^21^ ^]^ To further illustrate the advantages of our FeF_2_@PDC cathode in enhanced electronic and ionic conductivity, we conducted (Electrochemical impedance spectroscopy) EIS measurements (Figure S12, Supporting Information) which show that the FeF_2_@PDC electrode exhibits lower charge transfer resistance than the bare FeF2 electrode. Comparing our results to the state‐of‐the‐art FeF_2_ cathode studies (Table S1), our composite cathode shows the highest current density 60 C (34 A g^–1^) while maintaining an outstanding discharge capacity of 107 mAh g^–1^ (Figure 3c), which is in sharp contrast to most FeF_x_ cathodes that are hard to maintain an ideal capacity above 10 A g^–1^, thanks to the novel structural design of the composite cathode.

Noteworthy that we achieved a long cycle life of over 1900 cycles with a reversible capacity of over 500 mAh g^–1^ at 0.5 C (with >80% capacity retention), which is the longest cycle lifetime reported hitherto (Figure 3e). More details of the electrochemical performance in comparison with other studies are summarized in Table S2, Supporting Information. The corresponding voltage profiles show that the voltage hysteresis was larger in the initial cycles and gradually declined after 100 cycles (Figure 3d). The discharge plateau was obvious in the initial cycles and tended to be sloped afterward (Figure 3d). The formation of different intermediates may contribute to the sloping of discharge curves.^[^ ^17^, ^22^ ^]^

We also evaluated the long‐term cycling performance at 30 C and 60 C. The cells were pre‐cycled 10 cycles at 0.5 C to enable permeation of the electrolyte into the PDC. A discharge capacity of over 120 mAh g^–1^ with 60% retention at 30 C (17 A g^–1^) after 500 cycles, as well as a capacity of over 107 mAh g^–1^ at 60 C (34 A g^–1^) after 500 cycles were achieved (Figure 3f). To the best of our knowledge, this represents the longest cycle lifetime at such high current densities reported in FeF_2_. We attributed the excellent performance to the suppressed parasitic reaction by the formation of a nanometer thick Fe_3_O_4_ layer on the surface of the CAMs (which will be elaborated later), and again to the nanosize effect of FeF_2_ which permits rapid electron/ion transport. A smaller volume expansion (≈22%) during cycling, which close to its theoretical volume expansion and much lower than the experimentally observed volume expansion of bare FeF_2_ (41–57%),^[^ ^23^ ^]^ may also contribute to the superior performances (Figure S13, Supporting Information).

Presumably the parasitic reaction was largely avoided, the consumption of electrolyte would be minimized. Then the discharge and charge in diluted electrolyte should be doable if electrolyte was able to support sufficient Li+ transfer.^[^ ^24^ ^]^ The diluted LiFSI/DME electrolyte works for those materials with limited CEI formation.^[^ ^6^, ^7^ ^]^Thus, we tested the electrochemical performance of our composites in diluted LiFSI/DME electrolyte at 0.1 M, 0.5 M and 1 M, higher concentration of 3M was tested for comparison (Figure S14, Supporting Information). As we excepted, the cells show similar trend in long‐term cycling and rate test with no relation to electrolyte concentration. Note that excellent performance of FeF_2_@PDC in diluted electrolyte was also achieved, which has not been achieved before.

### 2.3 Postmortem Studies

**Figure 4:**
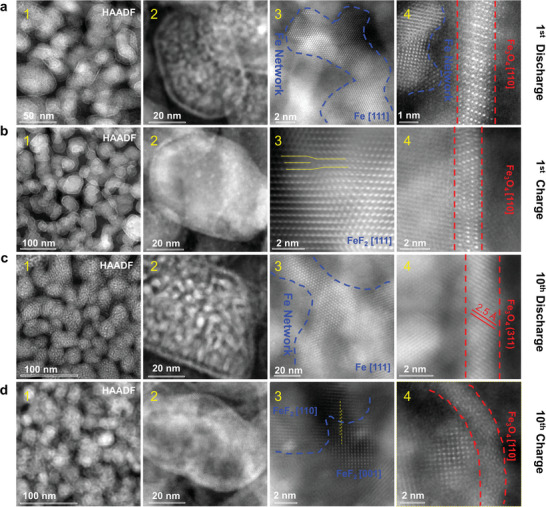
Ex situ HAADF‐STEM images illustrating the reversible electrochemical reactions and the formation stable Fe_3_O_4_ layers. a), The 1st discharge. a1,2), HAADF images showing that a Fe_3_O_4_ shell was formed on the outer surface of FeF_2_ and speckle‐like interconnected Fe particles are distributed inside the big particles due to the reduction of FeF_2_. a3), Atomic HAADF‐STEM image showing that Fe network was formed inside the big particle. a4), Atomic HAADF‐STEM image showing the formation of an about 2 nm thick Fe_3_O_4_ surface layer. b), The 1st charge. b1), HAADF images showing that the fine particles formed during discharge (a1,2) seem to have coalesced into a whole one with the shell remained intact. b3), HAADF‐STEM image of the particle interior demonstrating the formation a Fe network (a3) reconverted to FeF_2_ with dislocation. b4), HAADF‐STEM image showing that an about 2 nm thick Fe_3_O_4_ surface layer still remained after charge. c), The 10th discharge and d) the 10th charge. Similar to the first cycle, an Fe network was formed inside the individual big particles (c3), and an about 2 nm‐thick Fe_3_O_4_ was formed on the surface after discharge (c4). The Fe_3_O_4_ layer remained unchanged (d3), and the Fe network reconverted back to FeF_2_ (d4). Note that FeF_2_ particles with varying orientations were observed after the 10th charge.

To further elucidate the long cycling stability mechanism, we conducted ex situ TEM and HAADF‐STEM on the samples after the 1st and 10th charge/discharge cycles. Ex situ TEM images and the corresponding selected area electron diffraction (SAED) patterns show that FeF_2_ converted to tiny Fe particles after discharging and reconverted to FeF_2_ after charging (Figure S15, Supporting Information). Low magnification HAADF image (**Figure** **4**a1) indicates that the discharged particles remained intact without any fracture or pulverization. Higher magnification HAADF image (Figure 4a2) shows speckle‐like tiny nanoparticles dispersed in each individual big particle, and each big particle is surrounded by a dark shell followed by a bright shell on its surface. Atomic‐resolution HAADF image shows the Fe network formed inside the big particle (Figure 4a3 and Figure S16, Supporting Information). In the HAADF images, Fe nanoparticles displays brighter contrast as compared to LiF, as the intensity of a HAADF image is roughly proportional to Z^2^, where Z is the atomic number.^[^ ^25^ ^]^ HAADF and ABF images (Figure S17, Supporting Information) further show that the interconnected Fe nanoparticles formed a network with LiF interlaced in between. This observation is consistent with the proposed lithiation mechanism of FeF_2_.^[^ ^26^ ^]^ The crystallinity of LiF formed via lithiation remains controversial: with both crystalline^[^ ^8^, ^26^ ^]^ and disordered LiF^[^ ^26b^ ^]^ being reported. LiF is extremely beam sensitive,^[^ ^23^ ^]^ we thus conducted Cryo‐TEM (90 K) on the lithiated sample, which shows that the Fe network was surrounded by amorphous LiF (Figure S18, Supporting Information). Disordered LiF may enhance ionic conductivity compared to the high crystallized LiF, thus the former may promote the Li+ transport.^[^ ^20a^ ^]^ A closer view of the shell demonstrated that the crystalline structure of the shell matches excellently with that of Fe_3_O_4_ (Figure 4a4 and Figure S19, Supporting Information). During the 1st charge, the Fe and LiF were reconverted back to FeF_2_ (Figure 4b and Figure S20, Supporting Information). Interestingly, dislocations appeared in the charged FeF_2_ (Figure 4), which were rarely observed. Dislocation accumulation may lead to nanocrystallization of FeF_2_ during cycling. The Fe_3_O_4_ shell remained during charge with no visible change in thickness (Figure 4b4 and Figure S21, Supporting Information). After 10 cycles, the morphology of Fe and LiF composite was similar to that after the 1st discharge (Figure 4c), demonstrating the high reversibility of the conversion reaction. The FeF_2_ was reconverted after the 10th charging (Figure 4d). Based on the TEM and HAADF‐STEM results, the conversion reaction of FeF_2_ can be written as

(1) $$\mathrm{Fe}F_{2}+2Li^{+}+2e^{-}↔2\mathrm{LiF}+\mathrm{Fe}$$

Note that FeF_2_ with size of 5 nm and with different orientations was observed inside the particle after the 10th charge (Figure 4d3 and Figure S22, Supporting Information). This is possibly resulted from the electrochemical induced grain refinement: namely, during discharge, large FeF_2_ particles are refined into smaller Fe domains; during charge these small Fe domains are converted back to FeF_2_, leading to the formation of smaller FeF_2_ grains. Noteworthy the Fe_3_O_4_ shell was robust and stable during cycling, and its morphology did not change significantly after 10, 100 or even 1000 cycles (Figure 4c4, d4, Figures S23 and S24, Supporting Information). Note that the discharge voltage of Fe_3_O_4_ is below 1 V (vs Li+/Li).^[^ ^27^ ^]^ Therefore, Fe_3_O_4_ did not participate in the electrochemical reaction but it did play critical roles in conducting Li+, preventing the deleterious cathode/electrolyte interfacial reactions and dissolution of CAMs.

Apparently the Fe_3_O_4_ shell is critical to the achievement of excellent electrochemical performance. We suggest that the Fe_3_O_4_ shell was formed due to oxidation of Fe formed after the first discharge. To test this hypothesis, cryo‐TEM (90 K) with vacuum transfer capability was used to characterize the fully discharged FeF_2_ nanoparticles without PDC. The results show that the discharged particles were surrounded by a Fe_3_O_4_ layer, which was deduced from the lattice fringes with an interplanar distance of 2.50 Å that well matches the (311) plane of Fe3O4 (Figure S25, Supporting Information). The elemental mapping further confirms that the surface shell consists of Fe and O (Figure S26, Supporting Information). Therefore, we conclude that the Fe_3_O_4_ shell was formed from the electrochemical reaction rather than an artifact due to sample preparation. The formation of Fe_3_O_4_ was possibly caused by the reaction between the reduced Fe with the residual O in PDC, or with the O from the initial permeated electrolyte. Regardless, the O will be captured by the Fe atoms extruded from the first discharge. The mechanical strong and dense Fe_3_O_4_ with significantly decreased catalytic activity may have passivated the CAMs, thus hindered detrimental electrolyte decomposition and leaching of CAMs. Note that even in high concentration (3M) LiFSI/DME electrolyte, the CEI layer formed outside the Fe_3_O_4_ layer was unstable during cycling (Figure S27, Supporting Information). Moreover, an oxide layer can also form even in low concentration electrolyte (0.1 mol) (Figure S28, Supporting Information). These results further assert that it is the Fe_3_O_4_ layer rather than the CEI plays a significant role in stabilizing the cathode/electrolyte interface.

**Figure 5:**
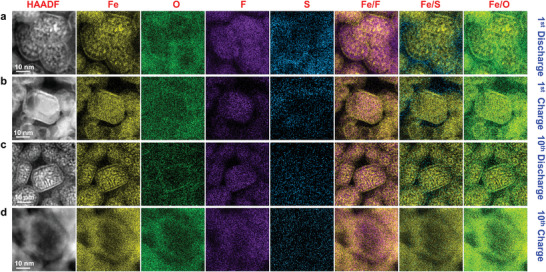
High‐resolution EDX elemental mapping illustrating structure evolution of the FeF_2_@PDC composite cathode. Note the thin Fe_3_O_4_ surface layer was stable during cycling. a) HAADF and corresponding element mapping after the 1st discharge showing that the O rich shell formed on the outer layer, which traced well with the Fe mapping in the same position. Besides, enrichment of S element can be seen outside the Fe layer, which was resulted from decomposition of LiFSI. b) The 1st charge. The whole elemental distribution is similar to that after the 1st discharge, except that the Fe became more uniform in the big particle due to the reformation of FeF_2_. c) The 10th discharge and d) the 10th charge. The Fe, O and F distributions are similar to that in the 1st discharge and charge cycle, respectively. Note that S element is evenly distributed out of the active materials, and there is no obvious aggregation outside the Fe‐O layer, indicating that the S‐rich products produced by the decomposition of LiFSI in the first cycle were unstable, and decomposition of LiFSI was suppressed in the subsequent cycles after a stable Fe‐O layer was formed.

To further rationalize the outstanding electrochemical performance, high resolution EDX analysis was conducted on the charged/discharged samples after the 1st and 10th cycles (**Figure** **5**). The HAADF images with the corresponding elemental mapping show that the Fe and fluorine (F) were well matched with a visible Fe network and uniform distribution of F. The O rich shell was observed in the outer layer and well traced the Fe mapping in the similar position, confirming again the formation of an oxide layer with Fe and O species. The sulfur (S), which was a major element of LiFSI, was accumulated out of the Fe_3_O_4_ shell, indicating decomposition of LiFSI in the first discharge. The Fe/F, Fe/S and Fe/O merged images intuitively confirmed the elemental distribution (Figure 5a). In the 1st charge, the elemental distribution was similar to that after discharge, but the elemental Fe appeared to be more homogeneous in the whole particle due to the reformation of FeF_2_ (Figure 5b). In the 10th discharge/charge (Figure 5c,d), the Fe, O and F distribution was similar to that in the 1st discharge/charge cycle. However, the accumulation of S out of the Fe_3_O_4_ shell disappeared. This is strong evidence that the decomposition of LiFSI was suppressed after the formation of Fe_3_O_4_ shell in the 1st cycle. It is thus concluded that the passivation effects of the Fe_3_O_4_ shell played a key role in mitigating the parasitic cathode/electrolyte interfacial reactions.

### 2.4 In Situ TEM Studies of Fe_3_O_4_ Formation in Conversion Reaction

**Figure 6:**
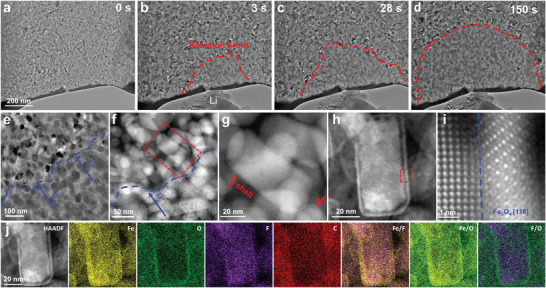
Structural evolution of the FeF_2_@PDC during in situ TEM lithiation. Note the formation of a Fe_3_O_4_ surface layer on individual particle after lithiation. a–d) Time‐lapse TEM images during lithiation at 0, 3, 28, and 150 s, respectively. e) TEM image of the reaction front (RF). f) Shells were formed on the surfaces of reacted FeF_2_ particles. g) A close view on a box outlined by red‐dashed lines in (f), indicating that the shell was formed at the beginning of lithiation. h) A shell formation on the particle surface. i) Close view of the red box in (h). j) EDX elemental mapping (h) confirmed the formation a uniform Fe_3_O_4_ shell on the surface of the particle.

Inspired by the HAADF‐STEM images (Figures 2 and 5), we attempted to clarify the formation mechanism of Fe_3_O_4_ layer by using in situ TEM technique.^[^ ^28^ ^]^ During discharge, a semi‐circular reaction front (RF) was emerged, which then propagated radially along the thin foil (**Figure** **6**a–d). The SAED patterns and the corresponding electron energy loss spectroscopy (EELS) confirmed the FeF_2_ in the nonreacted zone, and the formation of Fe and LiF after the conversion reaction (Figure 6e and Figure S29, Supporting Information). Interestingly, HAADF images revealed the formation of a Fe‐contained shell on the surface of individual FeF_2_ grains (Figure 6f–h), and the shell was proved to be Fe_3_O_4_ again (Figure 6,i and Figure S30, Supporting Information), which is consistent with the ex situ results obtained after cycling (Figure 4a4,c4). The formation of Fe_3_O_4_ can be attributed to O reacting with the highly reactive Fe atoms that was extruded from lithiation.^[^ ^8^, ^17^ ^]^ Note that the O source can be from the residual O in PDC or the trace O in the TEM column in the in situ results.^[^ ^29^ ^]^ A Fe deficit layer adjacent to the Fe_3_O_4_ layer was observed (Figure 6h), and similar results were observed in ex situ experiments (Figure 4a2,4c2), which is ascribed to the formation of LiF. And LiF is further demonstrated to be amorphous (Figure S31, Supporting Information). The corresponding elemental mapping shows that although the Fe and F mapping were well traced (Figure 6j), the typical Fe network was not observed (Figure 4a), suggesting that the lithiation process was just initiated, confirming that Fe_3_O_4_ was formed during the initial stage of lithiation.

## 3 Summary

By adopting a novel cathode design strategy, i.e., embedding FeF_2_ nanoparticles into a PDC (FeF_2_@PDC) matrix with excellent mechanical strength and mixed electronic/ionic conductivity, exceptional electrochemical performances were achieved in FeF_2_‐Li cells. The mechanisms leading to the exceptional performance were revealed at an atomic scale. Our major achievements are as following.

1. A record long cycle lifetime of 1900 cycles at 0.5 C with a reversible capacity over 500 mAh g^–1^ (>80% capacity retention) was achieved.
2. A record high rate of 60 C (discharge/charge in 1 min) with a reversible capacity of 107 mAh g^–1^ after 500 cycles was attained.
3. The in situ formation of a stable Fe_3_O_4_ layer on the surface of the FeF_2_ CAM prevented the deleterious CAM/electrolyte interfacial reactions and dissolution of active species.
4. The interconnected carbon network with mixed electronic/ionic conductivity in the PDC forms the charge transfer expressways for the CAMs with poor charge transfer kinetics.

This study sets new records in the performance FeF_2_ cathode and provides new understanding to the FeF_2_ electrochemistry, which will accelerate the development of high energy density FeF_2_ cathode‐based lithium batteries for electrical vehicle and grid energy storage applications.

## 4 Experimental Section

### Synthesis of FeF2@PDC Composite

In a typical synthesis process, excess amount of Fe powder (2.0 g, Sigma‐Aldrich, 99%) was reacted with 10 mL of H_2_SiF_6_ aqueous solution (25wt%, Sigma‐Aldrich) for 24 h. The mixture was then centrifuged to harvest the solids. The as‐received solids were then diluted to 20 mL with distilled water to get a FeSiF_6_ aqueous solution. 3 g PVP powder (Sigma‐Aldrich, average Mw ≈6500) was dissolved in 20 mL ethanol (Sigma‐Aldrich, 99.8%) and stirred for 2 h. Then the as‐prepared FeSiF_6_ and PVP alcohol solution was mixed and stirred for 2 h to obtain precipitates. Afterward, the precipitates were dried in oven under vacuum at 60°C overnight. To completely remove the solvent, the obtained powder was grinded and further dried at 150°C in vacuum. Finally, the powder was annealed at 260°C for 4 h followed by annealing at 550°C for 3 h under an argon flow. To obtain nano‐FeF_2_ powder without PDC, the FeSiF_6_ aqueous solution was dried and annealed at 260°C for 4 h under an argon flow, in which the following chemical reaction takes place: FeSiF_6_ → FeF_2_ + SiF_4_ (gas).

### Materials Characterization

The purity of as‐prepared FeF_2_@PDC composites was characterized by XRD using a Bruker D8 advance diffractometer in a transmission mode with Cu Ka X‐ray source operated at 40 kV and 200 mA at room temperature. The diffraction angles (2*θ*) were recorded from 10 ° to 80 ° with scanning rate of 2 ° min^−1^. XPS (ESCALAB 250Xi) was carried out to investigate the chemical state of the elements on the sample surface. TGA was performed on a thermal analyzer (STA 449 F5). To determine the carbonization temperature of PVP, TGA experiment was performed in argon atmosphere, and to measure the mass ratio of FeF_2_ in the FeF_2_@PDC composites, the TGA was performed in air. TEM and STEM samples were prepared by lift‐out via Ga^+^ ion‐beam milling in a FIB system (Helios G4 CX, Thermo Fisher Scientific). A protective thin Pt layer was deposited over the region of interest before milling. To minimize damage from electron beam and sufficiently thin the specimen for electron transparency, final milling was carried out at a voltage of ≈2 kV with a current of 44 pA. SAED and HRTEM were acquired in a Cs‐corrected environmental transmission electron microscope (ETEM, Titan G2, 300 kV, FEI). The HAADF‐STEM images and elemental mapping were acquired in a Cs‐corrected scanning transmission electron microscope (Titan cubed Themis Z 300 kV, Thermo Fisher scientific), which was equipped with a DCOR condenser lens spherical aberration corrector for forming a fine electron probe, and a quad‐silicon drift detector (Super‐X) optimized for rapid X‐ray collection. The collection semi‐angles of the STEM detectors were set to 65–200 mrad for HAADF imaging, and 8–17 mrad for ABF imaging. To avoid serious specimen damage and obtain reliable images, the beam current was adjusted to be as low as possible (20–40 pA). The HAADF and ABF image simulations were performed by xHREM program, which was based on the multislice method.^[^ ^30^ ^]^ The accelerating voltage, convergence, and collection angles used in the simulations are in agreement with the experimental values.

### Electrochemical Measurements

Slurry was made by mixing FeF_2_@PDC, PVDF binder and multiwalled carbon nanotubes (MWCNTs) (Sigma‐Aldrich, 98% carbon basis) with a weight ratio of 7:1:2 in N‐Methyl pyrrolidone (NMP) solvent (Sigma‐Aldrich, anhydrous, 99.5%). The resulted slurry was casted on aluminum foil via doctor blade and dried at 110°C in vacuum. Working electrodes were punched with a diameter of 11 mm and the average mass loadings of active materials were 1.2–1.5 mg cm^–2^. Batteries were assembled with 2032 stainless steel coin cell, with metal Li disk with a thickness of 0.5 mm as counter electrodes and Celgard 2400 (Celgard, USA) as a separator. Different mass ratio of LiFSI (Sigma‐Aldrich) salts were dissolved in DME (Sigma‐Aldrich) to form electrolytes with different concentration (0.1, 0.5, 1, 2, 3 M). The electrochemical performance was tested using battery testing system (LAND, CT3001A) over the voltage window of 1‐ 4 V (vs Li^+^/Li). All cells were tested at 25°C. EIS measurements were conducted on a Princeton electrochemical workstation with a frequency range from 1 MHz to 0.01 Hz.

### Postmortem Analysis

Coin cells were disassembled in a glove box and the electrodes were immersed in DME for 24 h to remove lithium salts. After the evaporation of DME in the glove box, the electrodes were transferred to FIB. In order to avoid air expose of the samples, a vacuum transfer device was used to transfer samples to the FIB‐SEM. In detail, the cycled cells were disassembled in a glove box filled with inert gas (Ar), then the electrode was placed in a vacuum transfer device in the glove box. The vacuum transfer device was then moved out and directly connected to FIB‐SEM system. Finally, the electrode was loaded under high vacuum into FIB for processing. During the entire sample preparation process, the samples were not exposed to air. Thin foil samples were processed for STEM observation. For Cryo‐TEM sample preparation, the cycled electrodes were washed with DME followed by ultra‐sonication in dimethyl carbonate (DMC) (Sigma‐Aldrich, anhydrous, 99%) solvent for 30 min. The samples dispersed in DMC were dropped onto a TEM micro‐grid in the glove box and frozen transferred to a Fischione MODEL 2550 cryo‐holder for observation without any exposure to air. All Cryo‐TEM images were acquired at low temperature (90 K) under low dose.

### In Situ TEM Observation

The pillar and sheet shape samples were fabricated from coarse cutting to fine polishing of FeF_2_@PDC by FIB under a beam current continuously decreasing from 21 nA to 14 pA. The as‐prepared pillars and sheets were used as working electrode with Li metal as counter electrode, and Li_2_O naturally formed on the surface of lithium metal was used as a Li^+^ ion conductive solid electrolyte. A bias of ‐0.5 V was applied between the two electrodes to drive migration of Li^+^ ions through the electrolyte.

## Conflict of Interest

The authors declare no conflict of interest.

## Author Contributions

Y.S and J. C. contributed equally to this work. Q.H. and J.H. conceived and designed the project. Y.S. synthesized the materials and carried out the electrochemical performance test. J.C., H.S., T.Y., Q.L., and C.D. carried out the EDS‐STEM and HAADF‐STEM experiments. H.L. and L.G. carried out the in situ experiments. H.S. carried out the HAADF and ABF image simulations. H.L., D.Z., X.Z., J.Z., and H.Y. carried out TEM and STEM samples prepared by lift‐out via ion‐beam milling in an FIB system. Y.S., J.C., B.G., X.L., Y.L., Y.G., J.Y., Y.L., and H.Q. contributed to the data analysis. Y.S., Q.H., and J.H. cowrote the manuscript with input from all the authors.

## Supporting information

Supporting Information

## Data Availability

The data that support the findings of this study are available from the corresponding author upon reasonable request.
