# Supplementary material for: Enabling Long Cycle Life and High Rate Iron Difluoride Based Lithium Batteries by In Situ Cathode Surface Modification
Source: Adv Sci (Weinh). 2022 May 14;9(21):2201419. doi: 10.1002/advs.202201419 (PMC9313485; doi:10.1002/advs.202201419)
Supplement: Supplementary file 1 — Supporting Information [file ADVS-9-2201419-s001.pdf]

Support Information

Enabling Long Cycle Life and High Rate Iron Difluoride Based Lithium Batteries by In-Situ Cathode Surface Modification

Yong Su[1], *Jingzhao Chen*[2], *Hui Li*[2], *Haiming Sun*[2, 3], *Tingting Yang*[2], *Qiunan Liu*[2], *Satoshi Ichikawa*[3], *Xuedong Zhang*[1], *Dingding Zhu*[1], *Jun Zhao*[2], *Lin Geng*[2], *Baiyu Guo*[2], *Congcong Du*[2], *Qiushi Dai*[2], *Zaifa Wang*[2], *Xiaomei Li*[2], *Hongjun Ye*[2], *Yunna Guo*[2], *Yanshuai Li*[2], *Jingming Yao*[2], *Jitong Yan*[2], *Yang Luo*[2], *Hailong Qiu*[2], *Yongfu Tang*[2], *Liqiang Zhang*[2], *Qiao Huang*[1]*, *Jianyu Huang*[1, 2]*

1 School of Materials Science and Engineering, Xiangtan University, Xiangtan, Hunan 411105, P. R. China.

2 Clean Nano Energy Center, State Key Laboratory of Metastable Materials Science and Technology, Yanshan University, Qinhuangdao 066004, P. R. China.

3 Research Center for Ultra-High Voltage Electron Microscopy, Osaka University, Ibaraki, Osaka 567-0047, Japan.

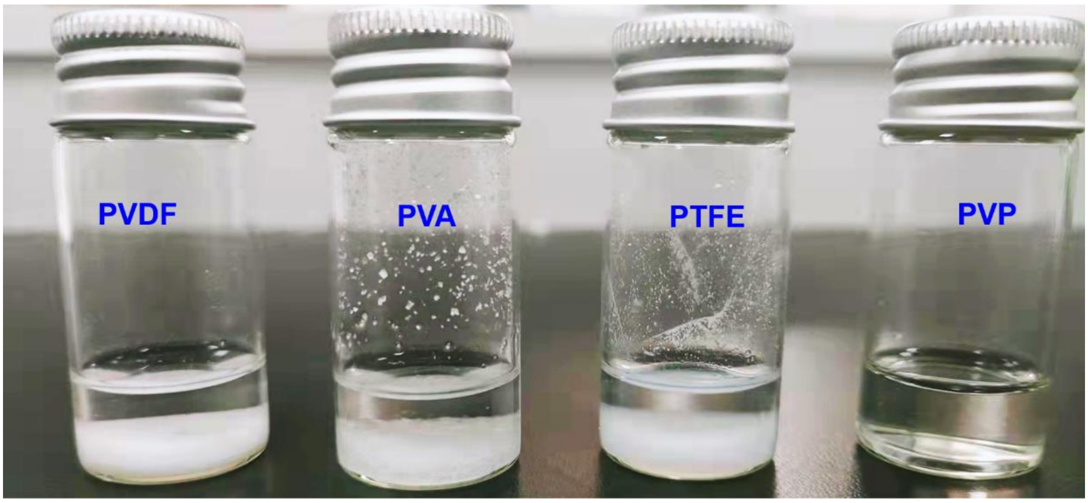

Figure S1. Solubility of different polymers in ethanol. It shows that only PVP can dissolve well in ethanol, resulting in a transparent solution.

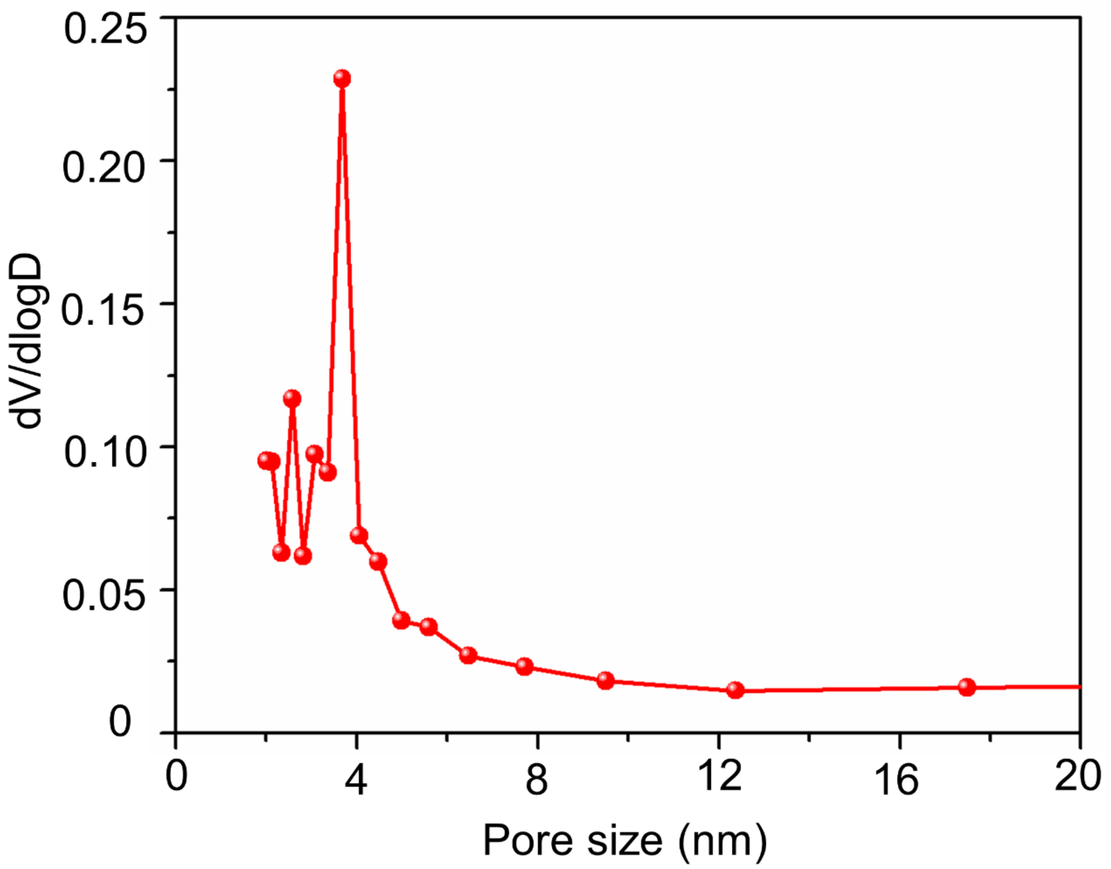

Figure S2. The pore size distributions of FeF_2@PDC.

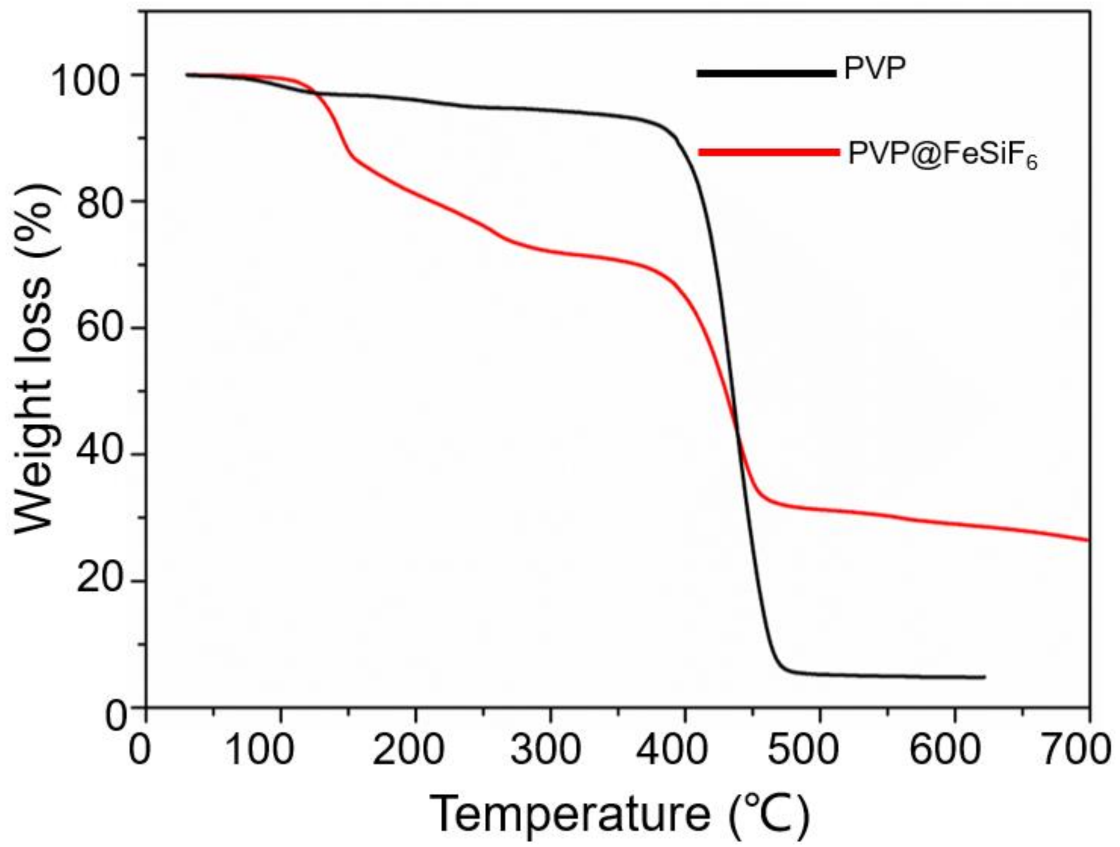

Figure S3. TGA analysis of the PVP, polymer and hydrated $FeSiF_6$ precipitates in Ar atmosphere showing that initial carbonization temperature was about 470 °C.

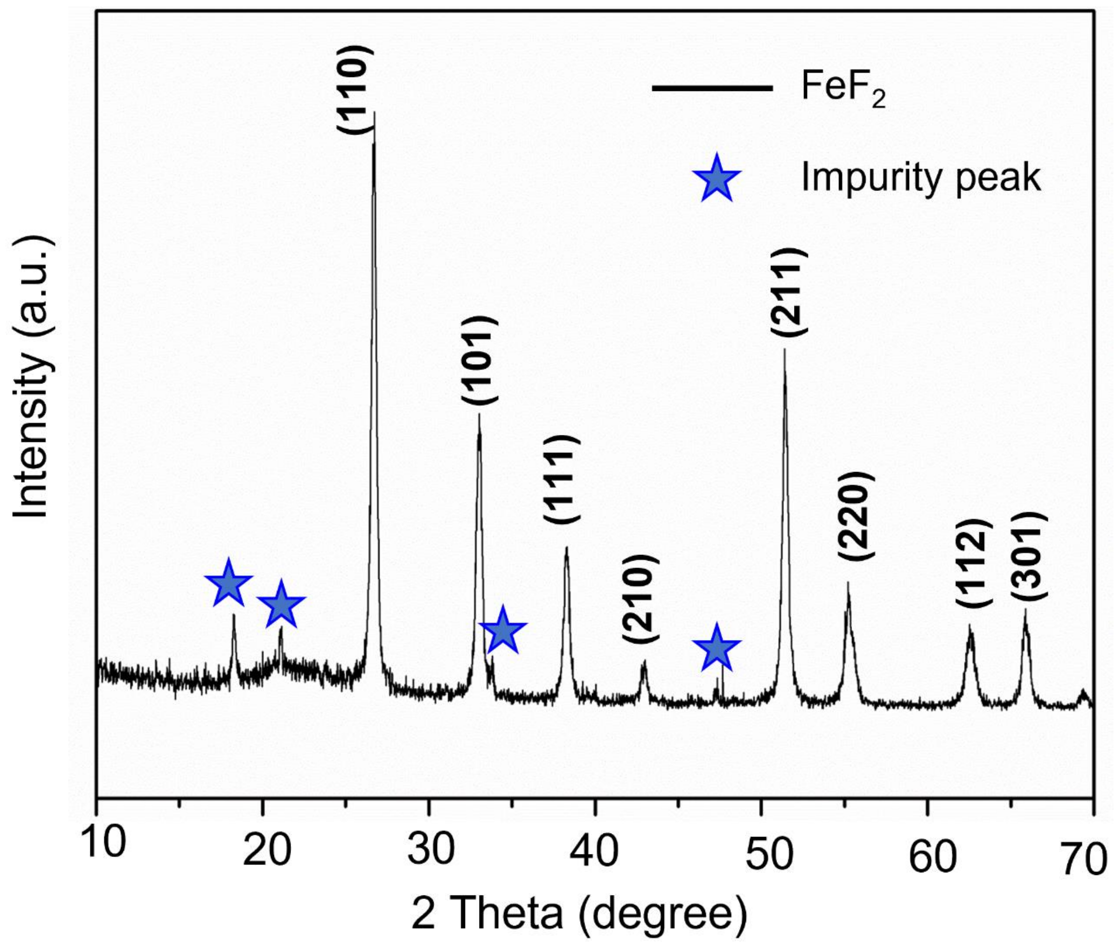

Figure S4. XRD of an FeF_2@PDC composite carbonized at 600 °C, showing the presence of undesirable side products.

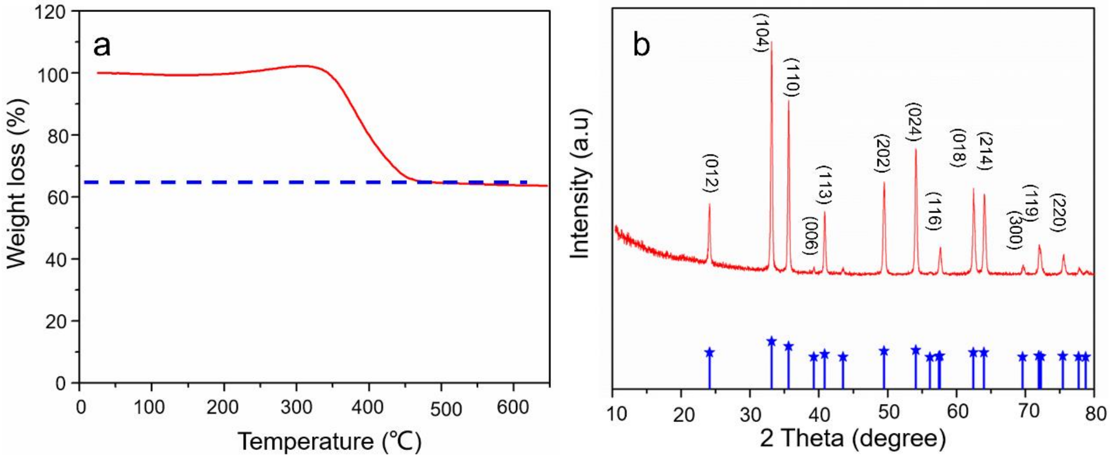

Figure S5. The FeF_2 content in FeF_2@PDC. (a) TGA analysis from room temperature to 650 °C in air atmosphere. (b) XRD of the product after TGA experiment. The results show that FeF_2 converted to α-Fe_2O_3 after TGA and the proportion of Fe_2O_3 in the composite was 65%. Based on the Fe element conservation (Fe_2O_3~$2FeF_2$), the proportion of FeF_2 in the composite was calculated to be 74%.

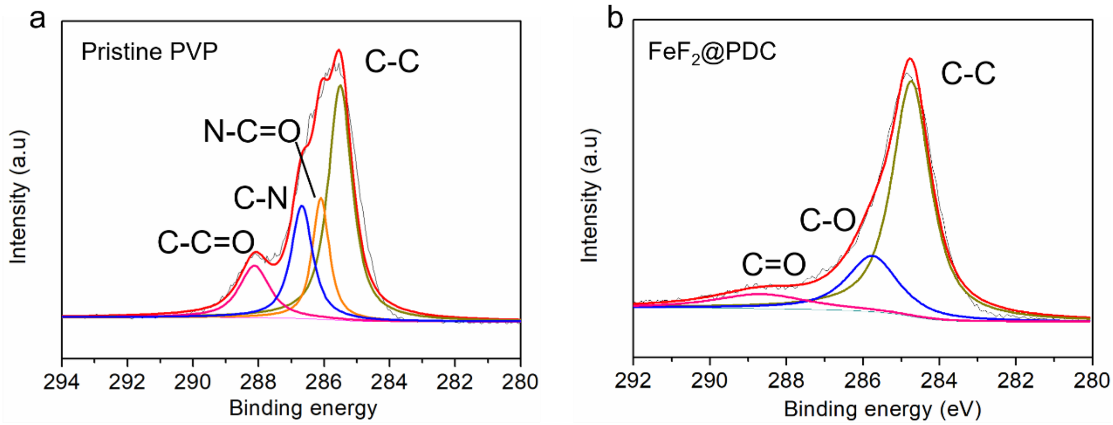

Figure S6 XPS spectra of C 1s from the pristine PVP (a) and FeF_2@PDC (b).

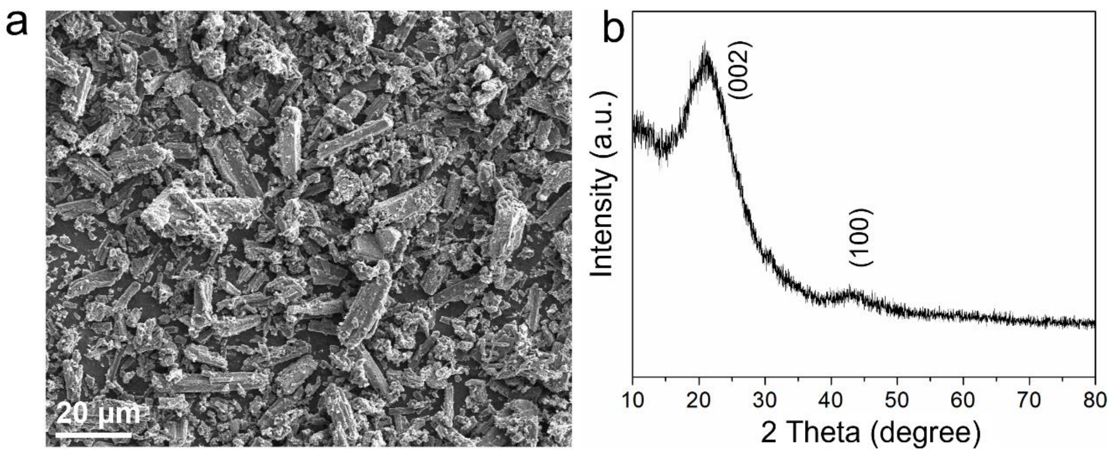

Figure S7. Characterization of PDC. (a) A SEM image showing the preserved PDC residue. The FeF_2@PDC composite was reacted with diluted hydrochloric acid to remove the FeF_2 particles. (b) The corresponding XRD of the PDC residue.

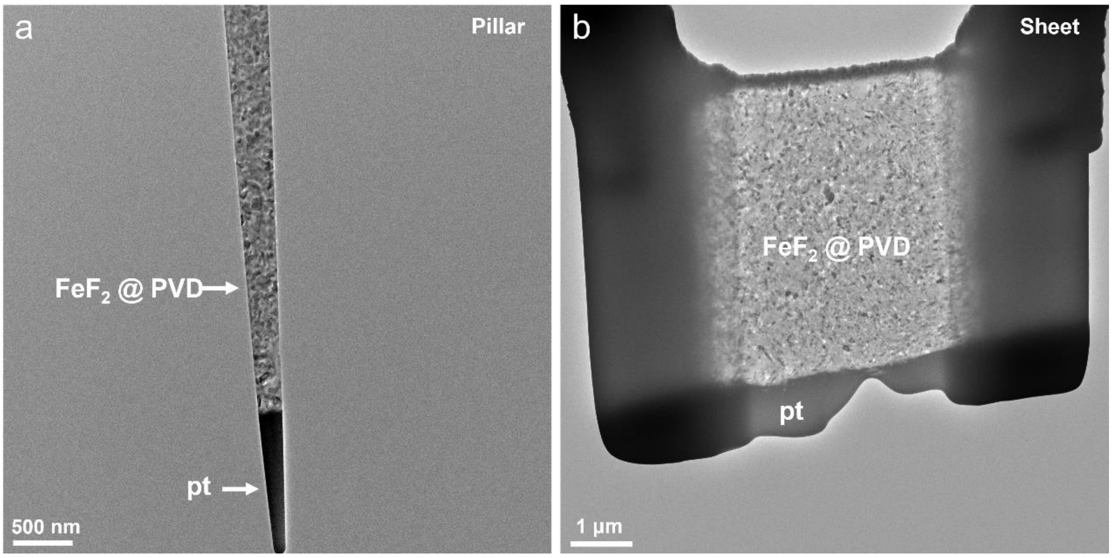

Figure S8. TEM sample preparations. Nano-rod (a) and thin foil (b) FeF_2@PDC samples were fabricated by focused ion beam (FIB) for electron microscopy investigations.

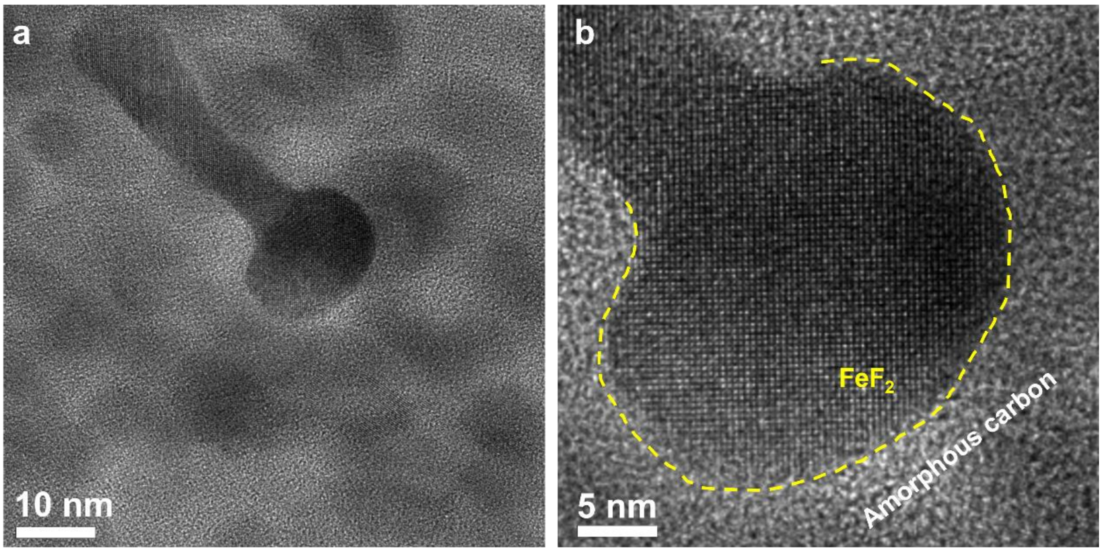

Figure S9. TEM and HRTEM characterization of the pristine FeF_2@PDC sample. (a) A TEM image showing clearly that FeF_2 particles are embedded in a carbon matrix. (b) A HRTEM image of a single FeF_2 nanoparticle, showing that crystalline FeF_2 is embedded in an amorphous carbon matrix, and the contact between the two is intimate.

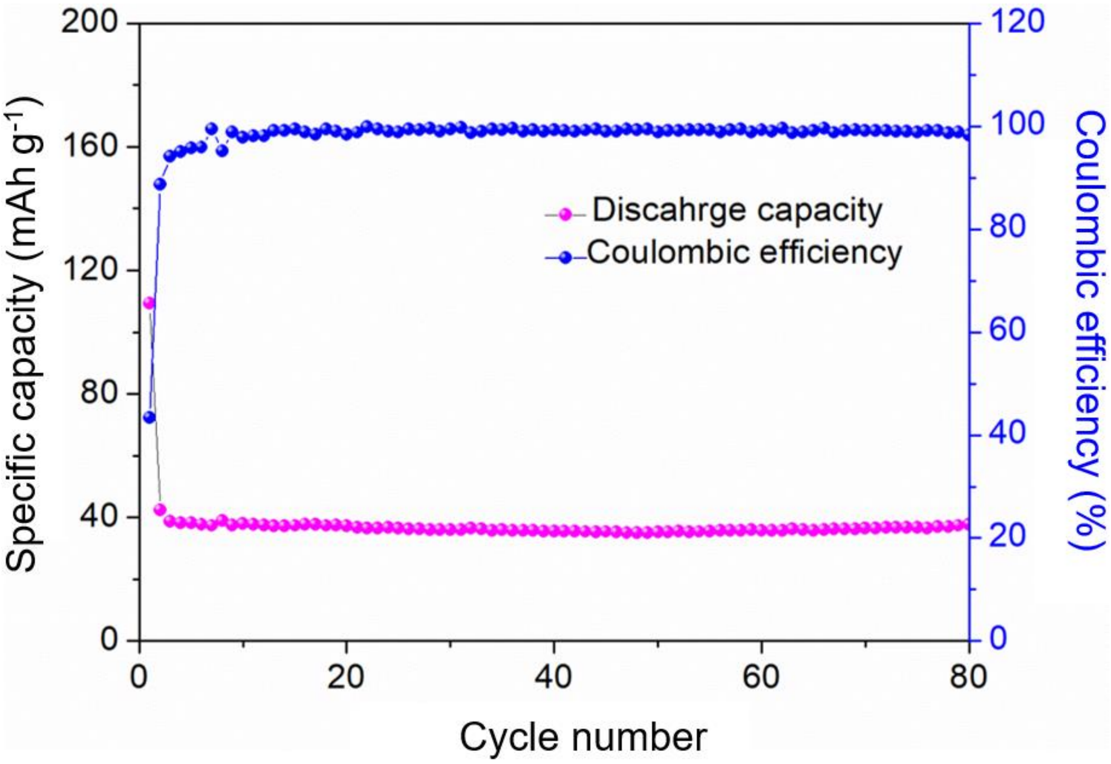

Figure S10. Cycling performance of bare PDC at 280 mA g^{-1} over a voltage window from 1 - 4 V (versus. Li^{+}/Li), showing a capacity of about 40 mAh g^{-1}.

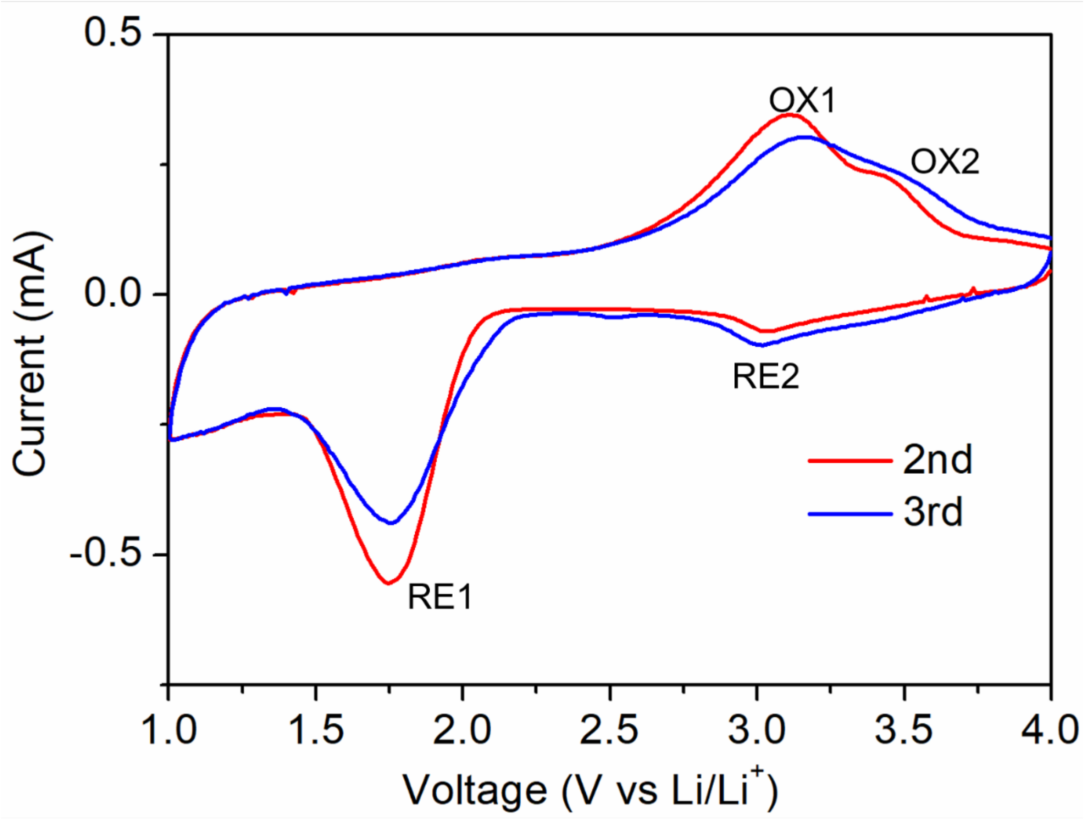

Figure S11. CV curves of the FeF_2@PDC-Li cell.

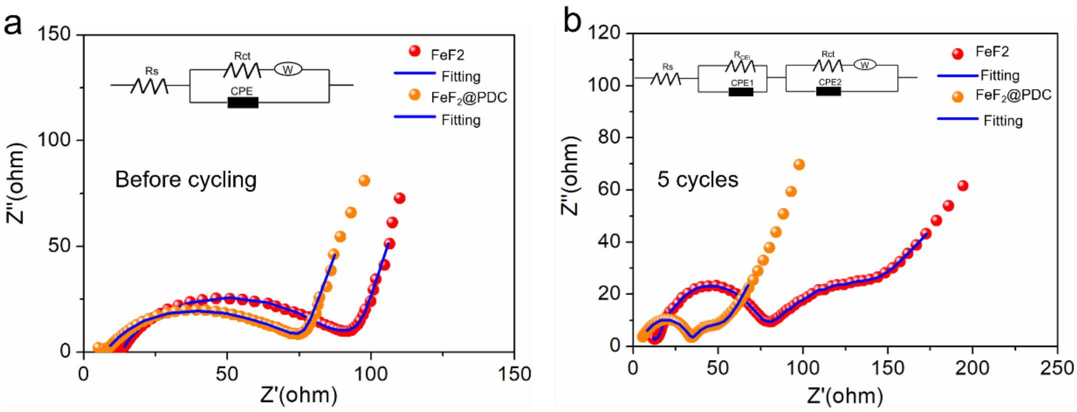

Figure S12. EIS results of the pristine FeF_2 and FeF_2@PDC before (a) and after 5 cycles (b).

Equivalent circuits used for fitting of the experimental data were provided on the top of the plots. The bare FeF_2 we used here was synthesized using the same method as the FeF_2@PDC cathode. The R_S represents the solution resistance mainly from the electrolyte. The R_{CEI} represents the diffusion resistance of Li^+ in the CEI layer (First semicircle at the lower frequency)[1]. The R_{ct} represents the electron/iron transfer resistance of the electrode from both the cathode/electrolyte interface and the bulk electrode (Second semicircle at the higher frequency). The CPE represents constant phase element for capacitance from the electric double layer of electrode/electrolyte interface (CPE2) and CEI (CPE1). The Warburg impedance (W) corresponds to the lithium-ion diffusion in the cell[2].

Before cycling, the resistance of the electrode mainly comes from the charge transfer resistance (R_{ct}) in the bulk electrode, which is determined by the electron/iron transfer of the electrode. Following the equivalent circuit, the R_{ct} for the bare FeF_2 is 78.3 Ω, which is close to the resistance of the FeF_2@PDC cathode (68.9 Ω) (Figure S12(a)). The slight R_{ct} difference before cycling does not affect the electrochemical performance since both of the R_{ct} are rather small.

However, when we cycled the cell for 5 cycles, the advantage of the FeF_2@PDC cathode emerged. As shown in Figure S12(b), After five cycles, the R_{ct} for bare FeF_2 increased from 78.3Ω to 157.6Ω, while that for FeF_2@PDC was only 30.9 Ω. This indicates the beneficial effect of the structure integrity and robustness of the FeF_2@PDC cathode in the charge and discharge process, with the electron/ion transfer pathways being effectively constructed. Note that for the bare cathode particles large volume changes during cycling will damage the microstructure thus ruins the electron/ion transfer pathways. Additionally, the R_{CEI} was 17.01 Ω for FeF_2@PDC cathode, and 62.04 Ω for the bare FeF_2. This is another strong evidence that the CEI formation in the FeF_2@PDC cathode was largely suppressed due to the embedding of FeF_2 into the PDC.

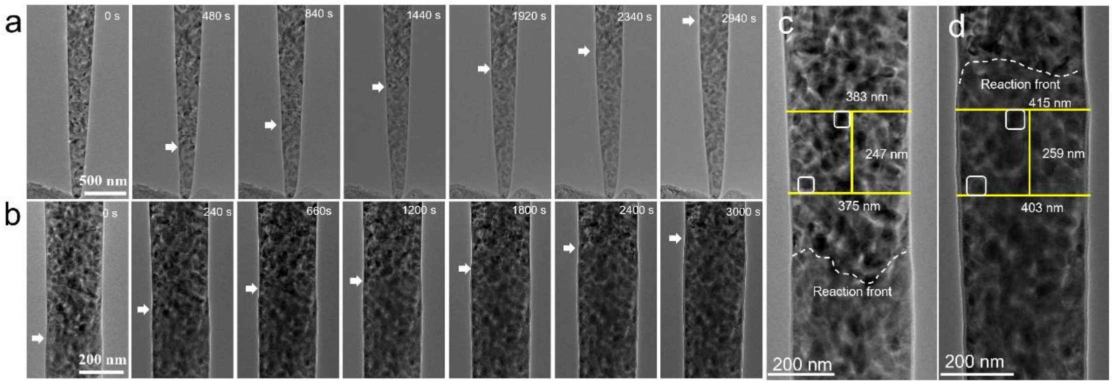

Figure S13. Assessing the volume expansion of the FeF_2@PDC composite cathode by in-situ TEM. (a) Time-lapse images showing the lithiation process. (b) Time-lapse images at high magnification showing the lithiation process. (c) and (d) are TEM images from the same location before and after lithiation, respectively. From these images, the volume expansion can be estimated. Assuming a tapered cone geometry, the volume is calculated by $V=1/3\pi h\ (R^2+Rr+r^2)$, where h is the height, R and r are the radius of the upper and lower cone, respectively. The parameters are: h = 247 nm, R = 383 nm, r = 375 nm for the pristine sample, and h = 259 nm, R = 415 nm, r = 403 nm after lithiation. The volume expansion can be obtained by: $(V_{reaction}-V_{origin})/V_{origin}$, where $V_{reaction}$ and V_{origin} are the volumes before and after lithiation, respectively. The volume expansion was calculated to be 22%, which is close to its theoretical volume expansion, but it is much lower than that reported in nanocrystalline FeF_2[3]. The structural integrity after lithiation of nano pillar testifies that the carbonized PDC acted like an elastic matrix to accommodate the volume changes of FeF_2, and thus maintain the structure integrity of FeF_2 in the long cycles.

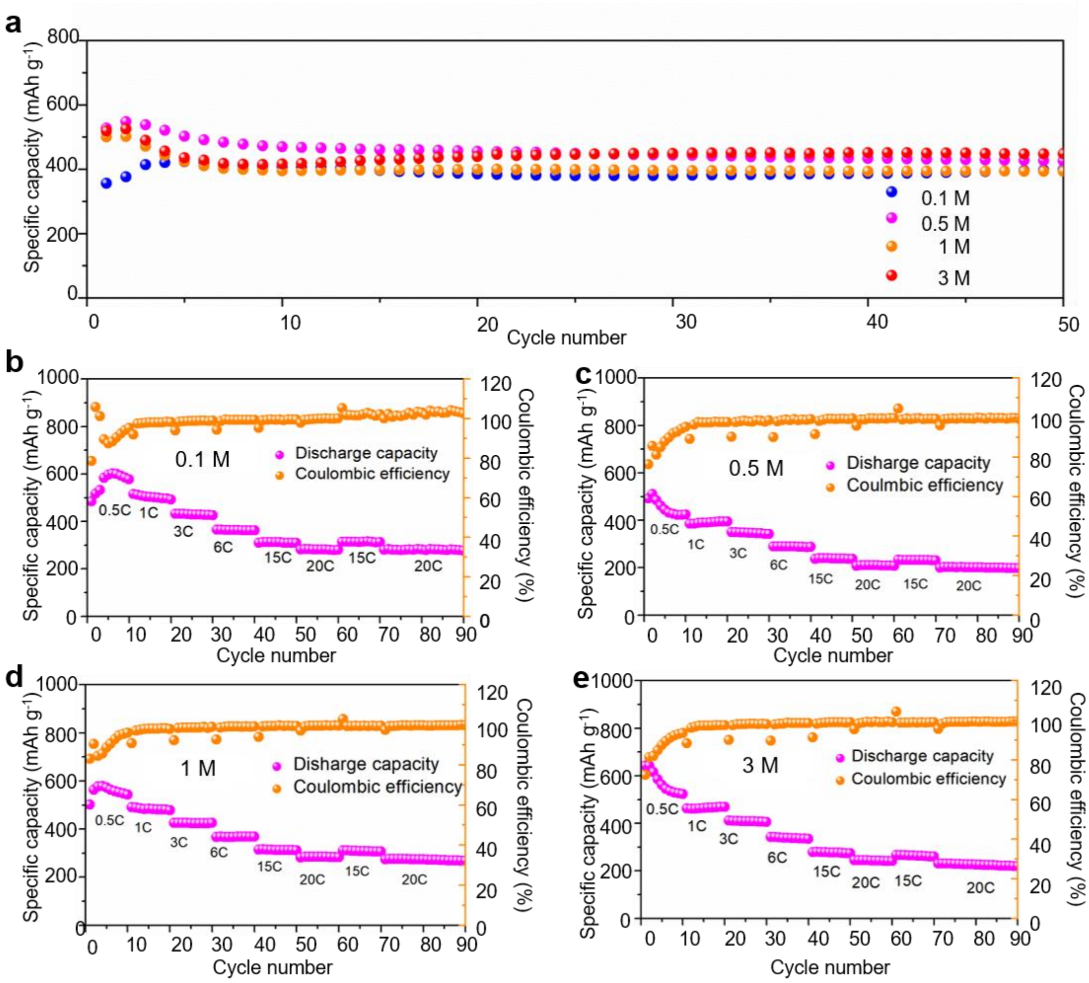

Figure S14. Electrochemical performances of FeF_2@PDC composite cathodes in different concentrations of electrolyte. (a) Cycling performances in 0.1 M, 0.5 M, 1 M and 3 M electrolyte at 0.5 C. Rate tests in (b) 0.1 M, (c) 0.5 M, (d) 1 M, (e) 3 M electrolytes. Note that no impressive cycling performance of FeF_2 in low concentration (<1 M) of LiFSI in DME has been reported due to the unstable CEI formation and the deterioration of ionic conductivity of the electrolyte[4]. The FeF_2@PDC composite cathode exhibits similar electrochemical performances in electrolytes with different concentrations, indicating a stable cathode/electrolyte interface was constructed, thus the electrolyte decomposition was mitigated.

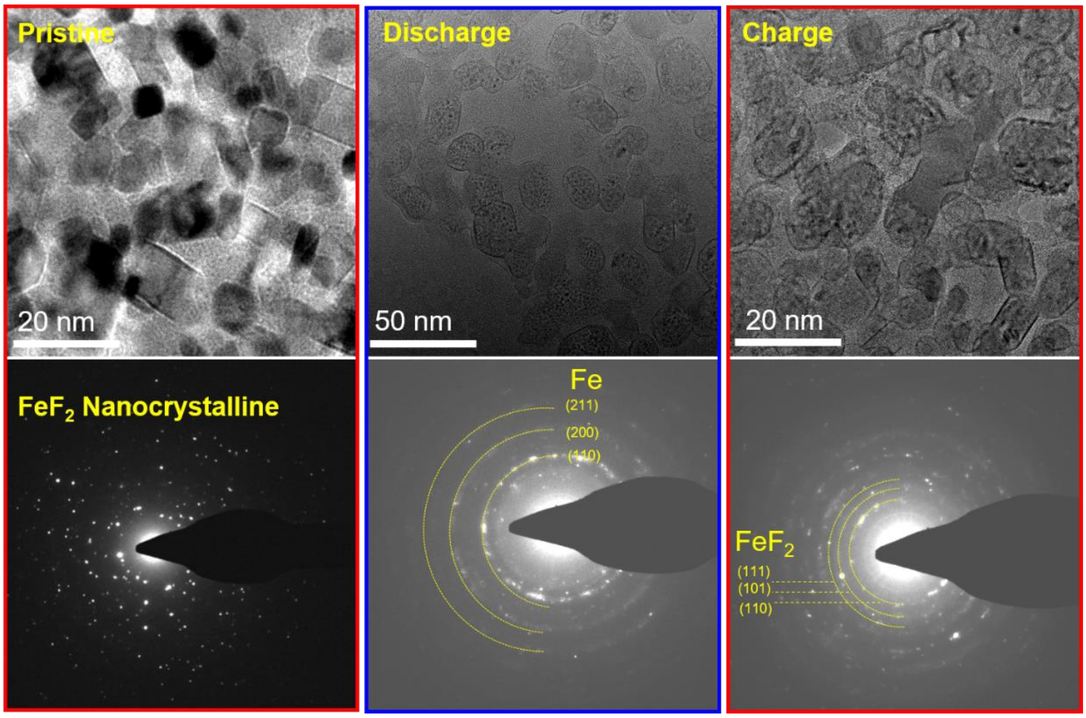

Figure S15. TEM images and the corresponding SAED of the pristine (left column), discharged (middle column) and charged FeF_2@PDC (right column).

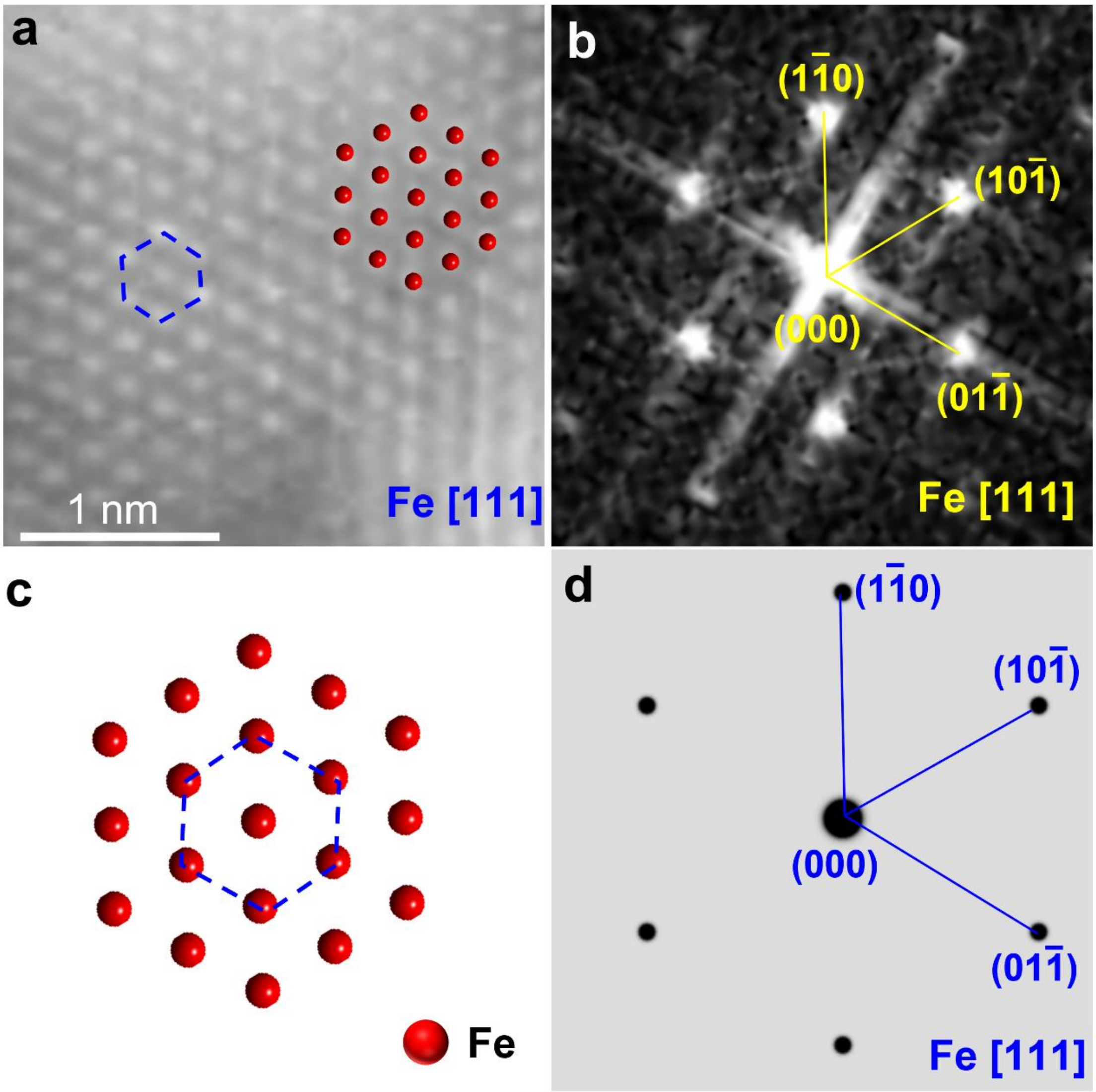

Figure S16. Atomic structure of Fe. (a) An atomic resolution HAADF-STEM image of Fe [111], and (b) the corresponding FFT from a FeF_2@PDC sample after the 1st discharge. (c) An atomic structure model of Fe, and (d) a calculated SAED from the structure model shown in (c). The simulated SAED (d) matches well with the experimental one (b).

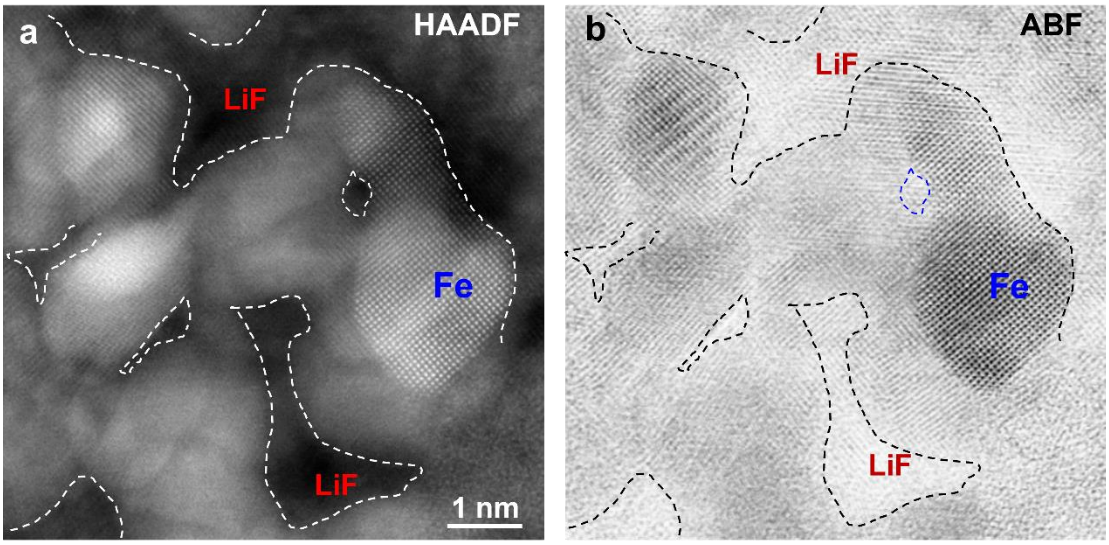

Figure S17. Atomic structure of the interconnected Fe network interwoven with amorphous LiF in a FeF_2@PDC sample after the 1st discharge. (a) A HAADF-STEM image, and (b) the corresponding ABF-STEM image.

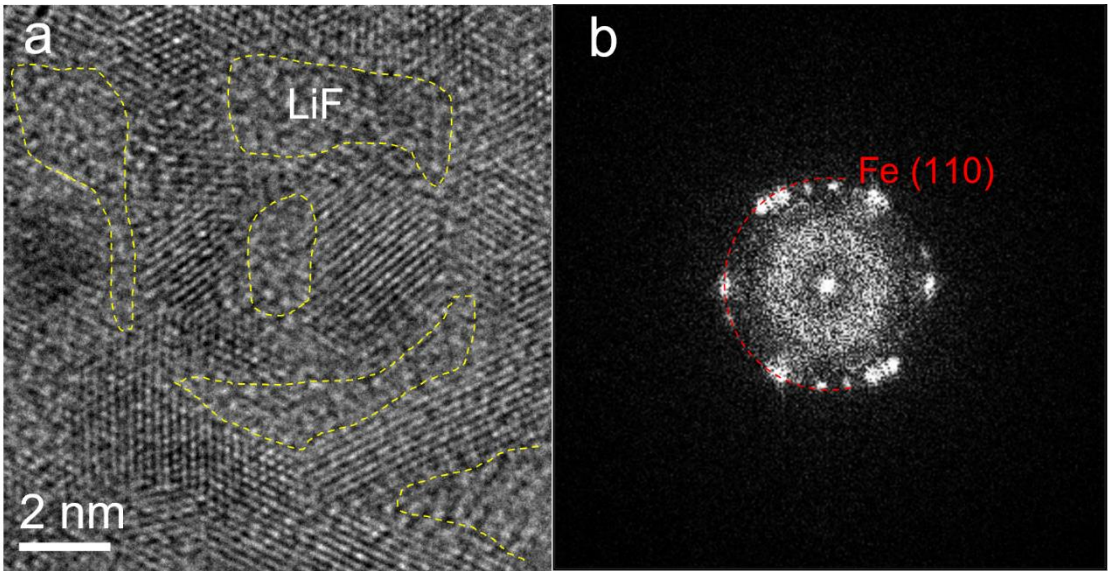

Figure S18. Cryo-TEM analysis of a bare FeF_2 nanoparticle after the 1st discharge. (a) A HRTEM image showing that the Fe network interwoven by amorphous LiF inside of particle. (b) FFT corresponding to (a).

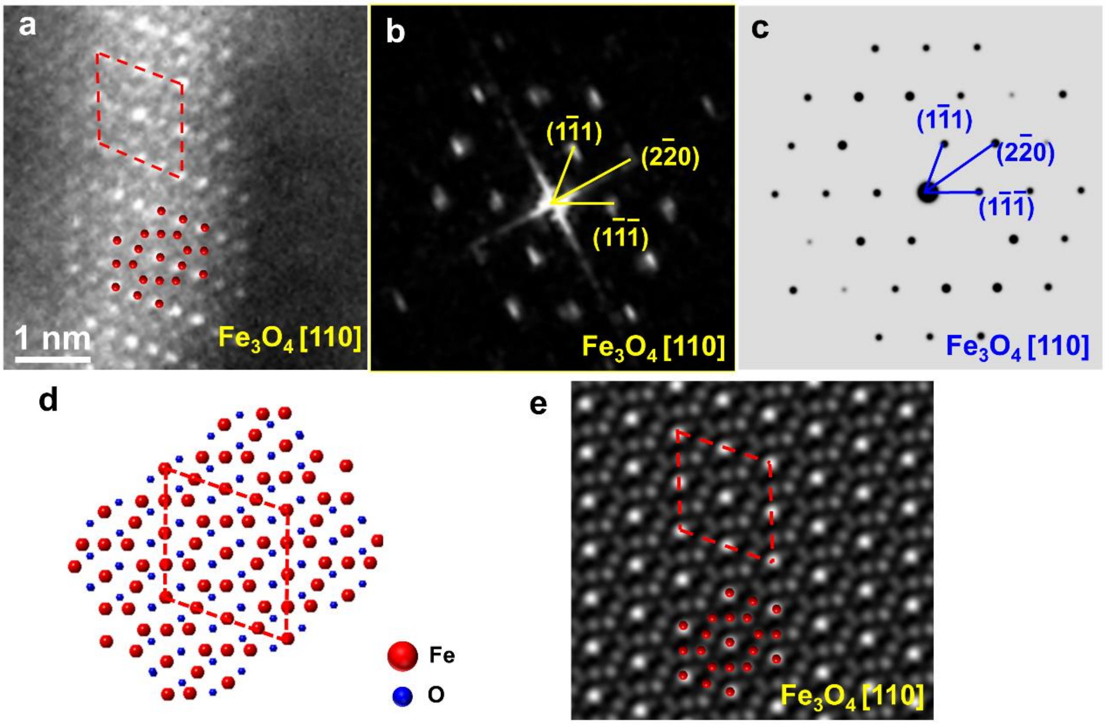

Figure S19. Atomic structure of the surface Fe_3O_4 layer. (a) An atomic resolution HAADF-STEM image of Fe_3O_4 [110], and (b) the corresponding FFT from a FeF_2@PDC sample after the 1st discharge. (c) A calculated SAED from the structure model shown in (d). The simulated SAED (c) matches well with the FFT shown in (b). (d) An atomic structure model of Fe_3O_4 [110]. (e) A simulated HAADF image based on the structural model shown in (d). The simulated HAADF image matches well with the experimental image in (a).

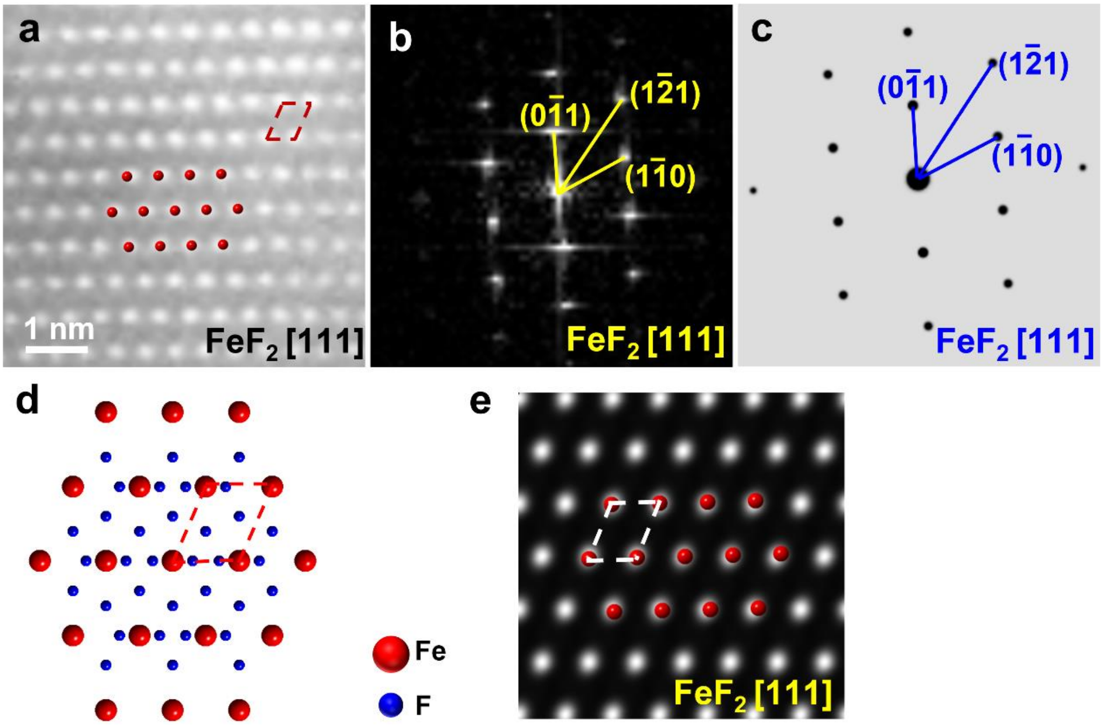

Figure S20. Atomic structure of FeF_2. (a) An atomic resolution HAADF-STEM image of FeF_2 [111], and (b) the corresponding FFT from a FeF_2@PDC sample after the 1st charge. (c) A calculated SAED from the structure model shown in (d). The simulated SAED (c) matches well with the FFT shown in (b)**.** (d) An atomic structure model of FeF_2 [111]. (e) A simulated HAADF image based on the structural model shown in (d). The simulated HAADF image matches well with the experimental image in (a).

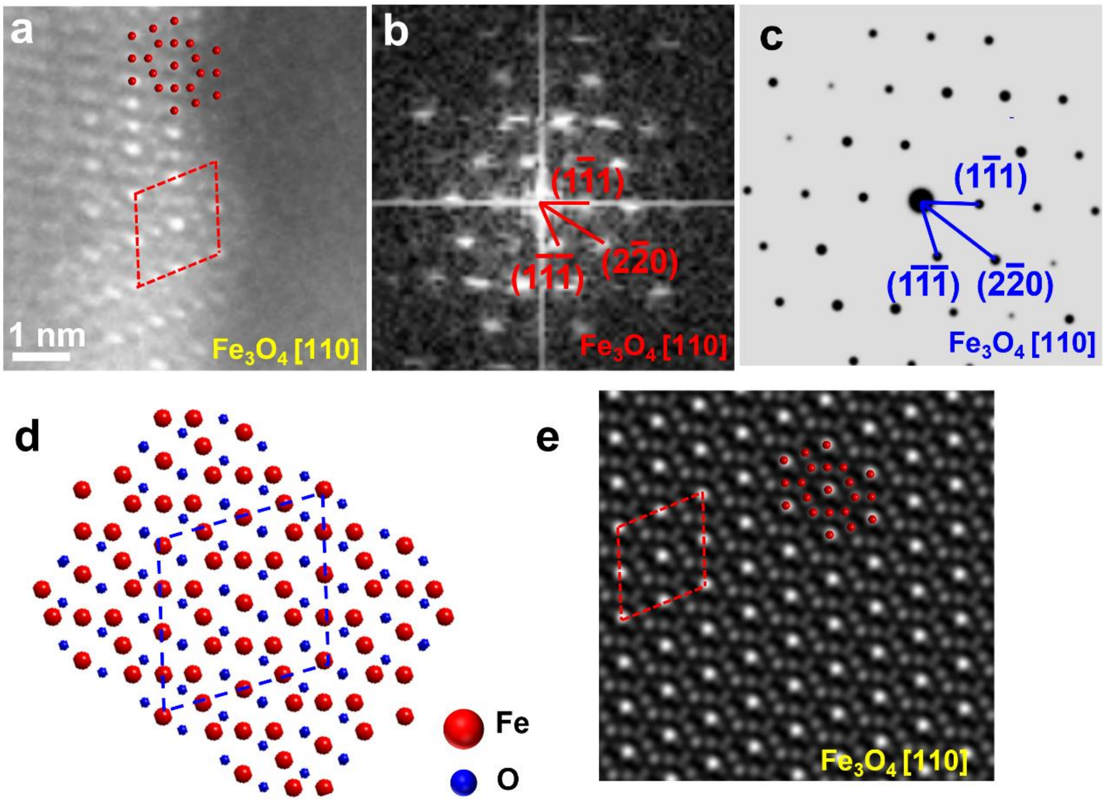

Figure S21. Atomic structure of the surface Fe_3O_4 layer. (a) An atomic resolution HAADF-STEM image of Fe_3O_4 [110], and (b) the corresponding FFT from a FeF_2@PDC sample after the 1st charge. (c) A calculated SAED from the structure model shown in (d). The simulated SAED (c) matches well with the FFT shown in (b). (d) An atomic structure model of Fe_3O_4 [110]. (e) A simulated HAADF image based on the structural model shown in (d). The simulated HAADF image matches well with the experimental image in (a).

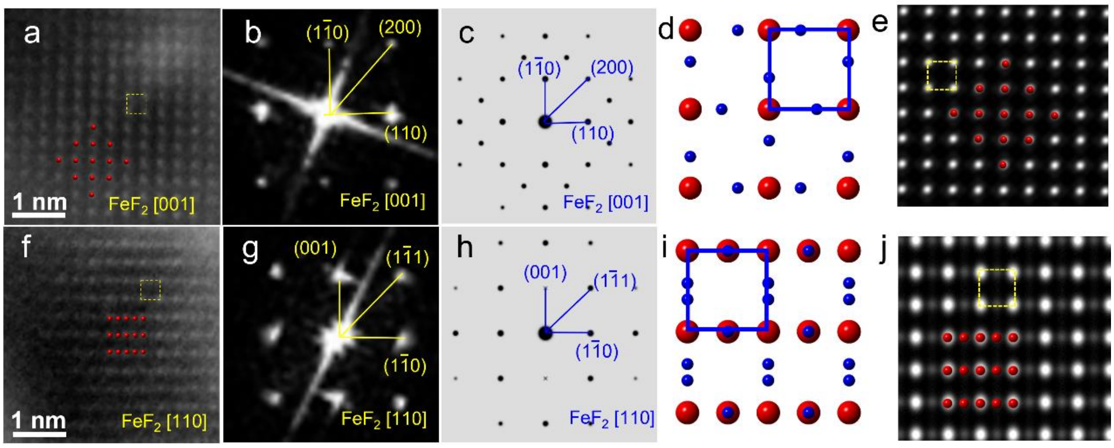

Figure S22. Atomic structure of FeF_2 obtained from an FeF_2@PDC sample after the 10th charge. (a) A HAADF-STEM image of FeF_2 along [001] direction, and (b) the corresponding FFT. (c) A simulated SAED from an atomic structure model shown in (d). (e) A simulated HAADF-STEM image of FeF_2 based on the structure model shown in (d), which matches well with the experiment image in (a). (f) A HAADF-STEM image of FeF_2 along [110] direction, and (g) the corresponding FFT. (h) A calculated SAED from an atomic structure model shown in (i). (j) A simulated HAADF-STEM image based on the structure model shown in (i), which agrees well with the experiment image in (f). FeF_2 with different orientation inside the big particle was possibly resulted from the accumulation of dislocations, which eventually led to the formation of new crystal boundary and thus the generation of sub-nano FeF_2.

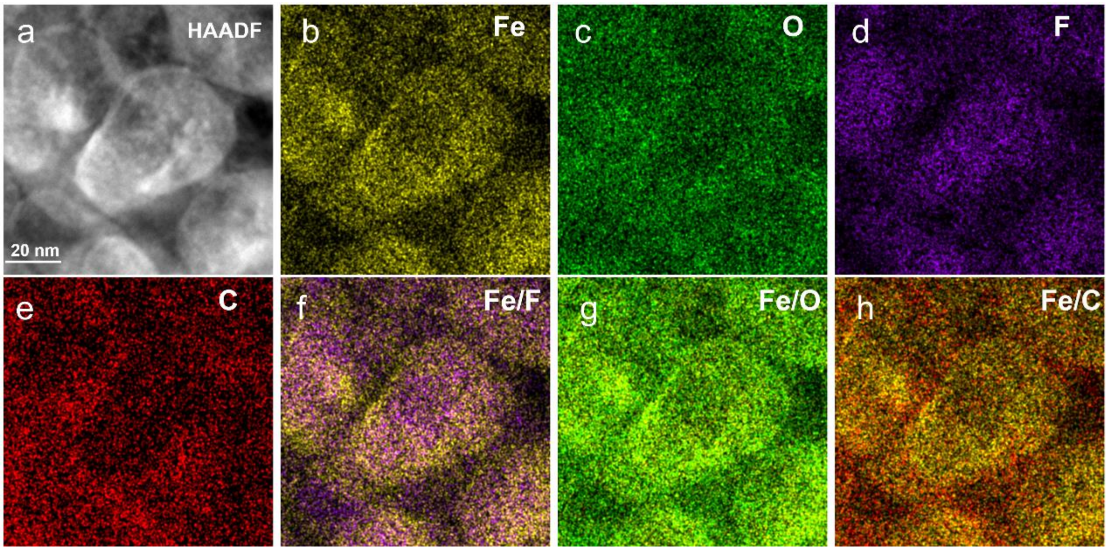

Figure S23. EDX elemental mapping of an FeF_2@PDC sample after 100 cycles showing that the oxide layer remained. (a) HAADF, (b) Fe, (c) O, (d) F, (e) C, (f)Fe/F merged, (g) Fe/O merged, (h) Fe/C merged elemental maps.

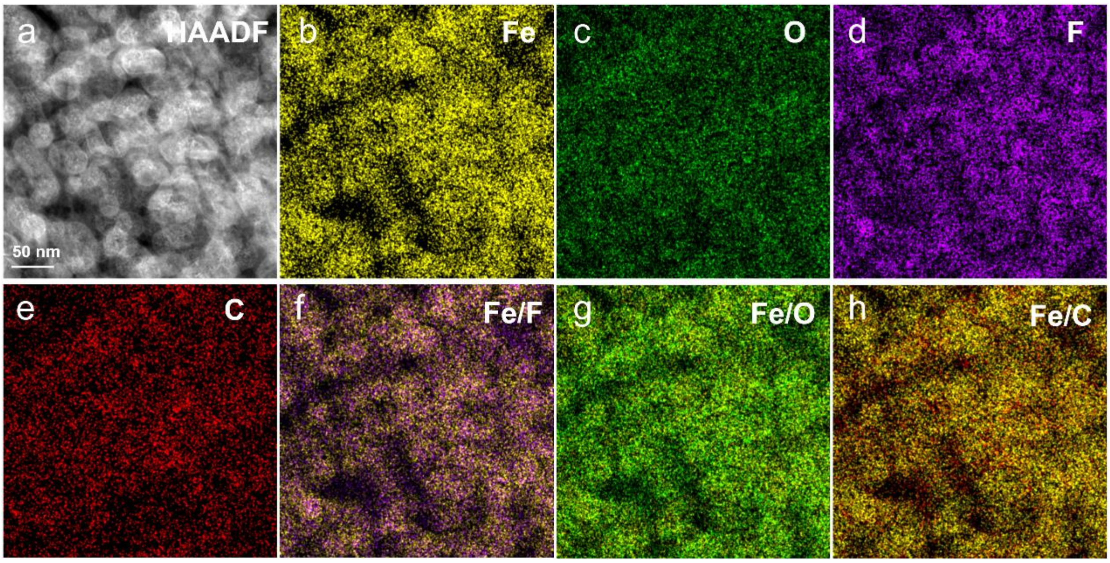

Figure S24. EDX elemental mapping of an FeF_2@PDC sample after 1000 cycles showing that the surface oxide layer remained. (a) HAADF, (b) Fe, (c) O, (d) F, (e) C, (f) Fe/F merged, (g) Fe/O merged, (h) Fe/C merged elemental maps.

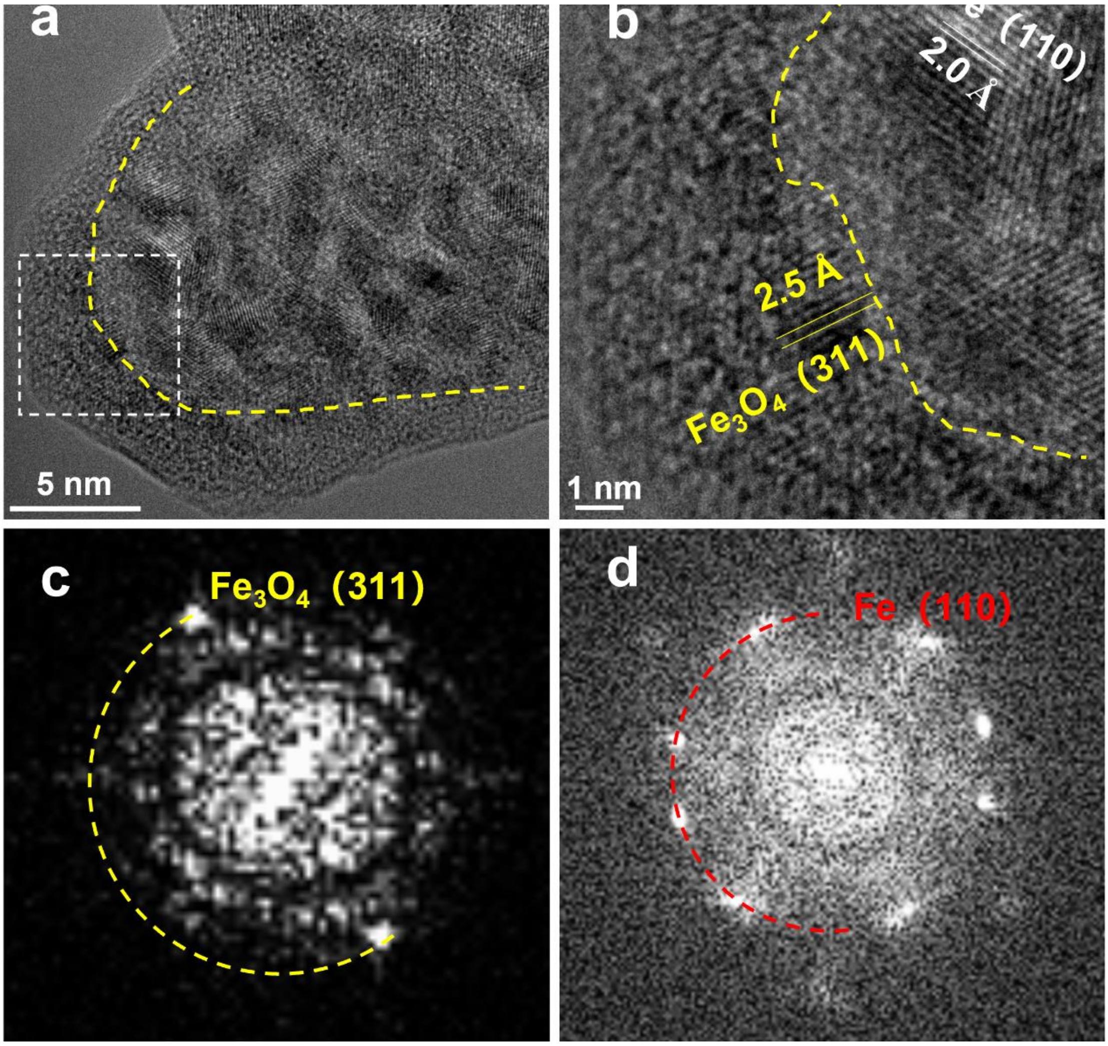

Figure S25. Cryo-TEM analysis of a bare FeF_2 nanoparticle after the 1st discharge showing that Fe_3O_4 was produced by electrolyte oxidation. (a) A HRTEM image of a single particle after discharging. Note the formation of interconnected Fe network interwoven with LiF, and a surface CEI layer. (b) A high magnification HRTEM image showing that the lithiated particle was surround by a shell. The lattice fringes with an interplanar distance of 2.50 Å can be ascribed to the (311) plane of Fe_3O_4 with Fe and amorphous LiF inside the shell. FFTs from (c) the surface and (d) the interior of the image shown in (b).

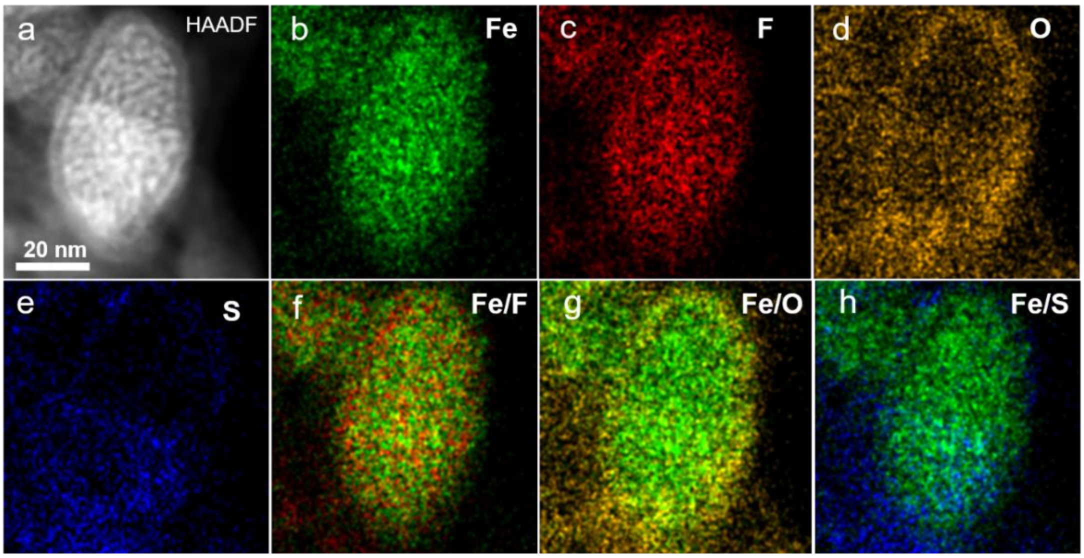

Figure S26. EDX elemental mapping from an FeF_2@PDC sample after the 1st discharge, showing the formation of a surface oxide layer. (a) HAADF, (b) Fe, (c) F, (d) O, (e) S, (f) Fe/F merged, (g) Fe/O merged, (h) Fe/S merged images.

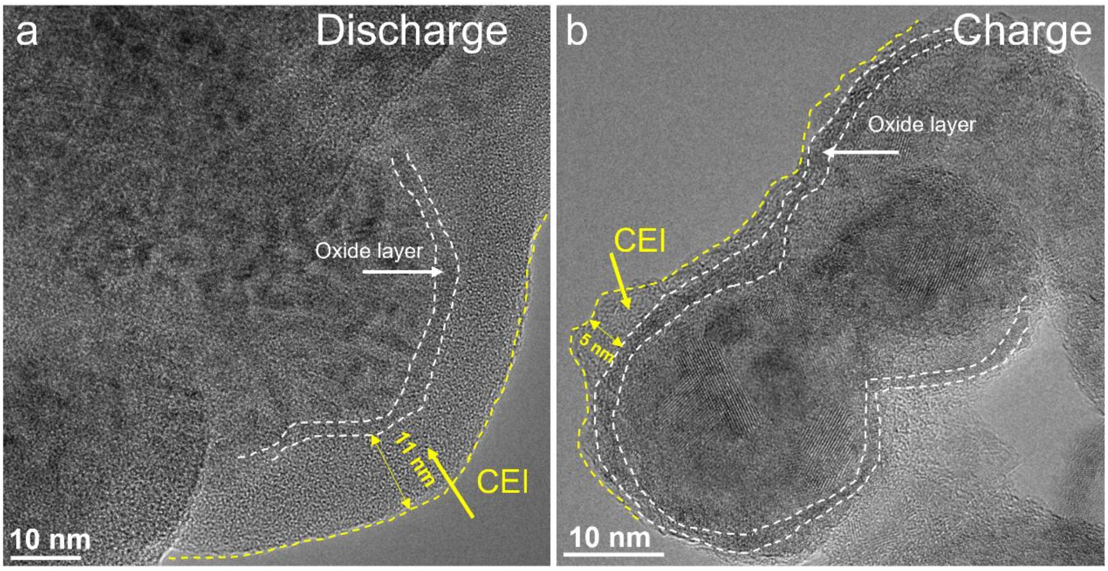

Figure S27. The CEI reformation on bare FeF_2 nanoparticles in a high molarity LiFSI/DME electrolyte (3 M). (a) A uniform CEI with a thickness of 11 nm was formed after the 1st discharge. (b) The CEI degradation occurred upon charging. The results suggest that CEI produced by decomposition of high concentration of electrolyte was also unstable during charge and discharge.

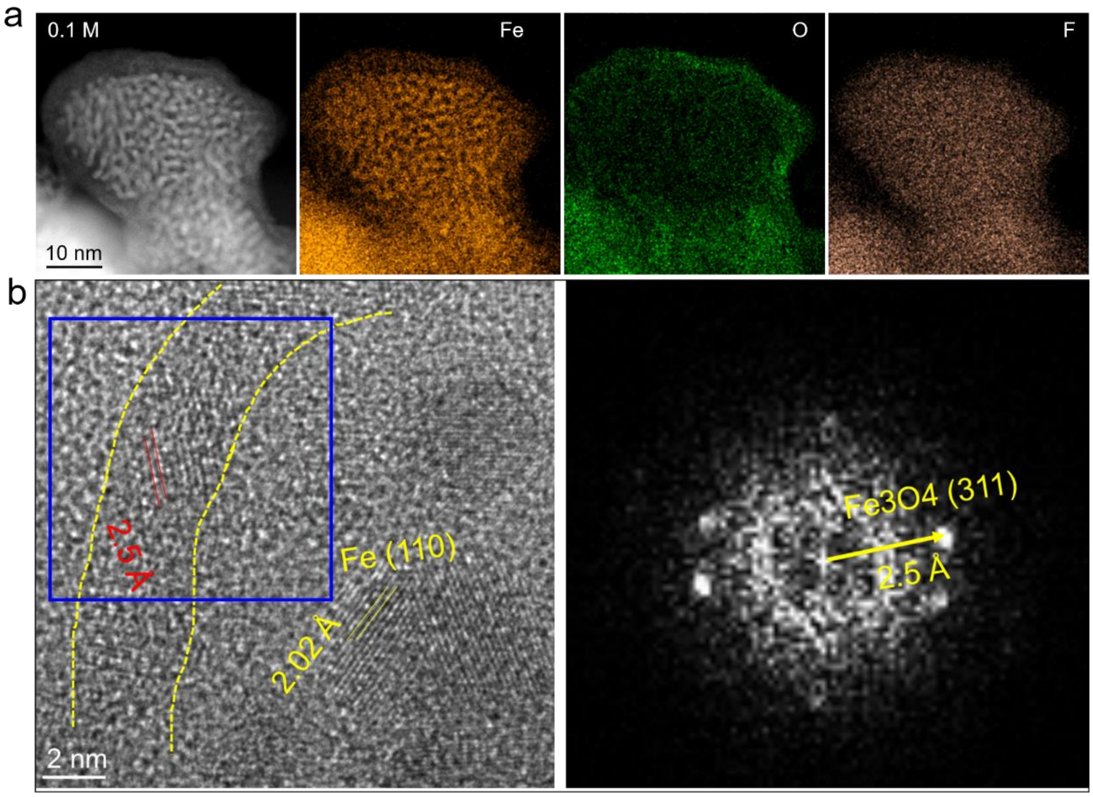

Figure S28 Electron microscopy characterization of FeF_2 cycled in 0.1 M LiFSI/DME electrolyte. A Fe_3O_4 layer was formed at the cathode/electrolyte interface. (a) A HAADF image and the corresponding EDX mapping. (b) A HRTEM image and the corresponding FFT.

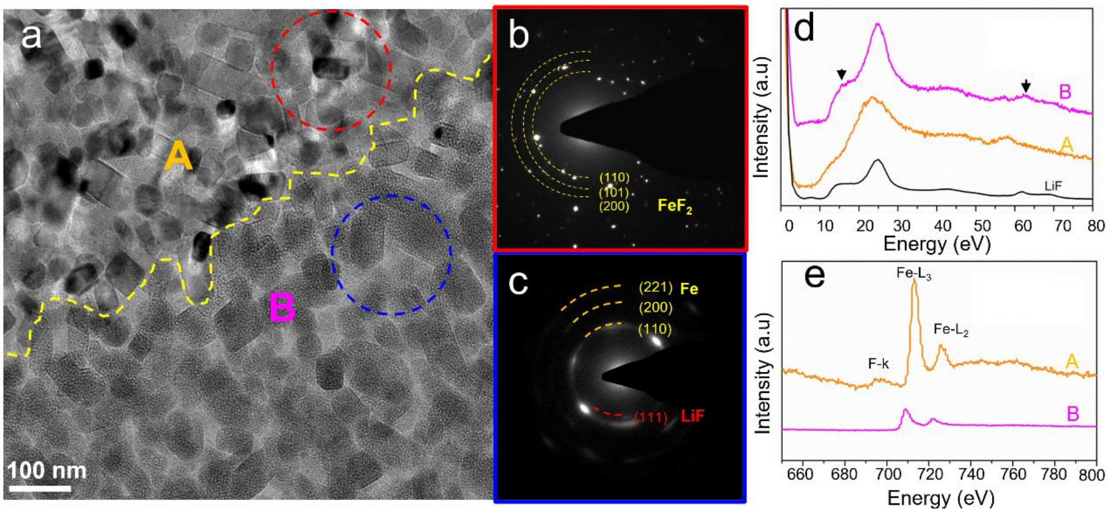

Figure S29. In-situ TEM lithiation of an FeF_2@PDC sample. (a) A TEM image showing a reaction front as marked by yellow dashed lines. (b) A SAED from an unreacted area (red dotted circle in (a)), and (c) a reacted area (blue dotted circle in (a)). Low-loss (d), and (e) core-loss EELS of an unreacted area (mark by "A" in (a)) and a reacted area (mark as "B" in (a)). The results show that FeF_2 converted to Fe and LiF after lithiation.

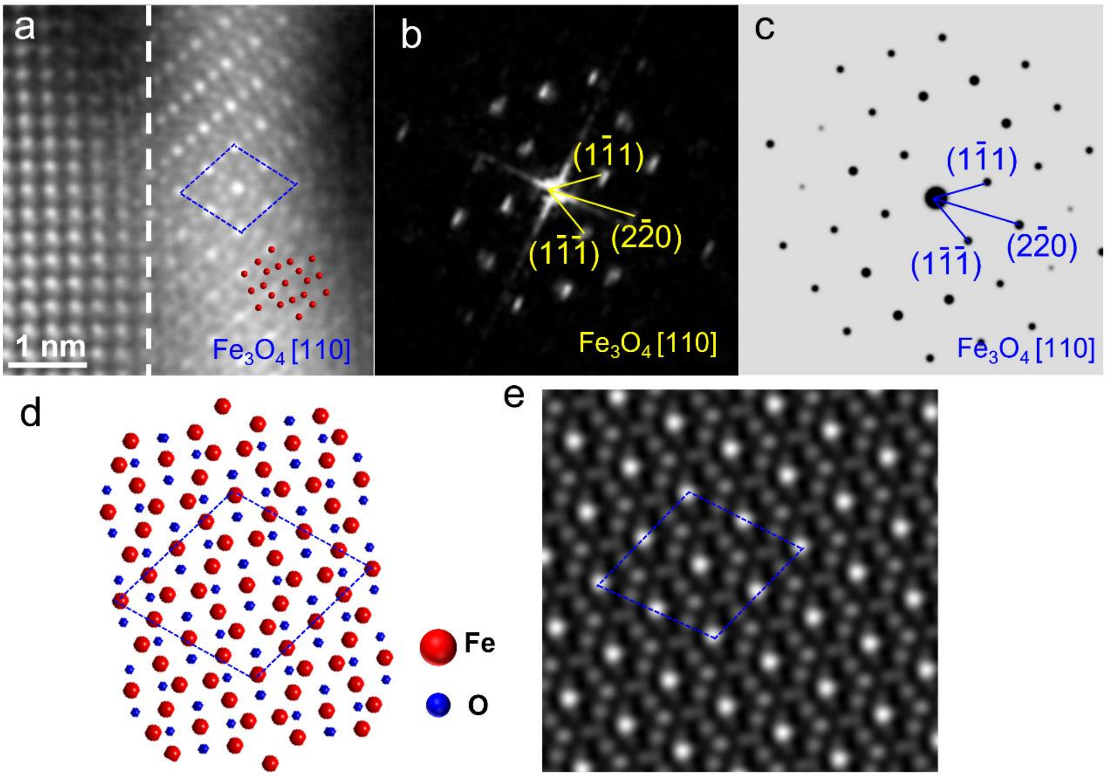

Figure S30. Atomic structure of the surface Fe_3O_4 layer from a FeF_2@PDC sample by in-situ lithiation. (a) A HAADF-STEM image of Fe_3O_4 along [110] direction, and (b) the corresponding FFT. (c) A calculated SAED from an atomic structure model shown in (d). (e) A simulated HAADF-STEM image of Fe_3O_4 [110] based on the structural model shown in (d). The simulated HAADF image fits well with the experimental image in (a).

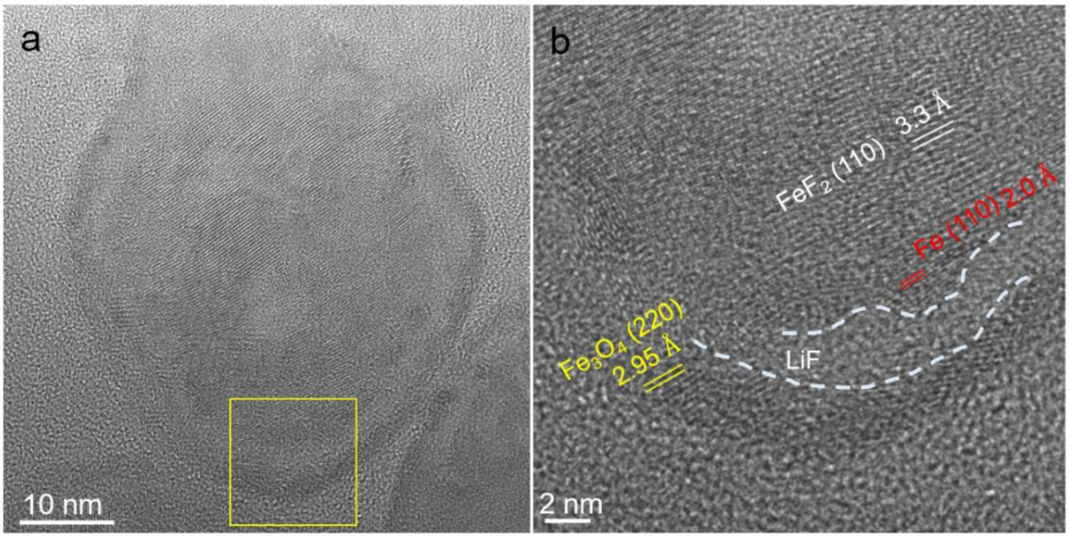

Figure S31. HRTEM image of an incomplete lithiated FeF_2 particle from a FeF_2@PDC sample after 1st discharge. (a) A HRTEM image showing the formation of a surface shell after lithiation. (b) A close view of a yellow boxed region in (a), showing an amorphous LiF layer underneath the surface the Fe_3O_4 layer.

Table S1. Comparison of rate performance of the previously reported FeF_x cathodes with this study

Sample	Highest rate (A/g)	Capacity (mAh/g)	Reference
**FeF_2@PDC**	**34**	**107**	**This work**
FeF_2@Al_2O_3	1	100	[5]
FeF_3@rGO	10	125	[6]
FeF_2@nanocrystal	0.28	500	[7]
FeF_2@nanorod	1	92	[8]
FeF_2@nanopores	0.57	125	[4a]

Table S2. Comparison of cyclic performance of the previously reported MF_x cathodes with this study

Sample	Cycle number	Current density (A/g)	Capacity (mAh/g)	Reference
**FeF2@PDC**	**1900**	**0.28**	**~500**	**This work**
FeF_2	1000	0.14	~300	[4a]
FeF_2	250	0.2	~150	[9]
FeOF	300	0.1	200	[10]
FeF_2	200	0.27	~300	[7]
FeF_2	100	0.28	~420	[4b]
FeF_2	50	0.2	~250	[5]
$FeF_3 \cdot 0.33H_2O$	1000	0.474	100	[11]
CoF_2	400	0.211	300	[12]
FeF_3	50	0.0237	~160	[13]
FeF_3	100	0.075	~100	[14]
$FeF_3 \cdot 0.33H_2O$	200	0.237	~175	[15]
FeF_3	1000	1.15	~125	[16]
CoF_2	200	0.1/0.5	330/200	[17]
FeF_2	50	0.08	~400	[18]
$Fe_{0.9}Co_{0.1}OF$	1000	0.5	~350	[19]
$FeF_3\ 0.33H_2O$	50	0.6	~130	[20]

References

[1] a)A. R. C. Bredar, A. L. Chown, A. R. Burton, B. H. Farnum, *ACS Appl Energy Mater* **2020**, 3, 66; b)M. G. S. R. Thomas, P. G. Bruce, J. B. Goodenough, *J Electrochem Soc* **1985**, 132, 1521.

[2] a)K. Chen, R. Pathak, A. Gurung, E. A. Adhamash, B. Bahrami, Q. He, H. Qiao, A. L. Smirnova, J. J. Wu, Q. Qiao, Y. Zhou, *Energy Storage Mater* **2019**, 18, 389; b)Y. Su, B. Fu, G. Yuan, M. Ma, H. Jin, S. Xie, J. Li, *Nanotechnology* **2020**, 31, 155401.

[3] F. Wang, H.-C. Yu, M.-H. Chen, L. Wu, N. Pereira, K. Thornton, A. Van der Ven, Y. Zhu, G. G. Amatucci, J. Graetz, *Nat Commun* **2012**, 3, 1201.

[4] a)W. Gu, O. Borodin, B. Zdyrko, H.-T. Lin, H. Kim, N. Nitta, J. Huang, A. Magasinski, Z. Milicev, G. Berdichevsky, G. Yushin, *Adv Funct Mater* **2016**, 26, 1507; b)Q. Huang, K. Turcheniuk, X. Ren, A. Magasinski, D. Gordon, N. Bensalah, G. Yushin, *Adv Energy Mater* **2019**, 9, 1803323.

[5] S. Kim, J. Liu, K. Sun, J. Wang, S. J. Dillon, P. V. Braun, *Adv Funct Mater* **2017**, 27, 1702783.

[6] J. Zhai, Z. Lei, D. Rooney, K. Sun, *Electrochim Acta* **2019**, 313, 497.

[7] A. W. Xiao, H. J. Lee, I. Capone, A. Robertson, T.-U. Wi, J. Fawdon, S. Wheeler, H.-W. Lee, N. Grobert, M. Pasta, *Nat Mater* **2020**, 19, 644.

[8] J. Zhou, D. Zhang, X. Zhang, H. Song, X. Chen, *ACS Appl Mater Interfaces* **2014**.

[9] H. Liang, Z. Hu, Z. Zhao, D. Chen, H. Zhang, H. Wang, X. Wang, Q. Li, X. Guo, H. Li, *Journal of Energy Chemistry* **2021**, 55, 517.

[10] W. Li, Y. Chen, A. Zangiabadi, Z. Li, X. Xiao, W. Huang, Q. Cheng, S. Lou, H. Zhang, A. Cao, X. Roy, Y. Yang, *ACS Appl Mater Interfaces* **2020**, 12, 33803.

[11] Q. Cheng, Y. Pan, Y. Chen, A. Zeb, X. Lin, Z. Yuan, J. Liu, *Inorg Chem* **2020**, 59, 12700.

[12] F. Wu, V. Srot, S. Chen, M. Zhang, P. A. van Aken, Y. Wang, J. Maier, Y. Yu, *ACS Nano* **2021**, 15, 1509.

[13] T. Kim, W. J. Jae, H. Kim, M. Park, J. M. Han, J. Kim, *J Mater Chem A* **2016**, 4, 14857.

[14] L. Zhang, S. Ji, L. Yu, X. Xu, J. Liu, *RSC Advances* **2017**, 7, 24004.

[15] L. Zhang, L. Yu, O. L. Li, S.-Y. Choi, G. Saeed, K. H. Kim, *J Mater Chem A* **2021**, 9, 16370.

[16] F. Wu, V. Srot, S. Chen, S. Lorger, P. A. van Aken, J. Maier, Y. Yu, *Adv Mater* **2019**, 31, 1905146.

[17] X. Wang, W. Gu, J. T. Lee, N. Nitta, J. Benson, A. Magasinski, M. W. Schauer, G. Yushin, *Small* **2015**, 11, 5164.

[18] J. Li, Y. Meng, Y. Wang, X. Li, Y. Lai, Y. Guo, X. Wen, D. Xiao, *Inorganic Chemistry Frontiers* **2021**, 8, 3273.

[19] X. Fan, E. Hu, X. Ji, Y. Zhu, F. Han, S. Hwang, J. Liu, S. Bak, Z. Ma, T. Gao, S.-C. Liou, J. Bai, X.-Q. Yang, Y. Mo, K. Xu, D. Su, C. Wang, *Nat Commun* **2018**, 9, 2324.

[20] B. Li, Z. Cheng, N. Zhang, K. Sun, *Nano Energy* **2014**, 4, 7.
